# A Subset of Roux-en-Y Gastric Bypass Bacterial Consortium Colonizes the Gut of Nonsurgical Rats without Inducing Host-Microbe Metabolic Changes

**DOI:** 10.1128/mSystems.01047-20

**Published:** 2020-12-08

**Authors:** Zhigang Liu, Isabelle Coales, Nicholas Penney, Julie A. K. McDonald, Jutarop Phetcharaburanin, Florian Seyfried, Jia V. Li

**Affiliations:** a Department of Metabolism, Digestion and Reproduction, Faculty of Medicine, Imperial College London, South Kensington Campus, London, United Kingdom; b Department of Brain Sciences, Faculty of Medicine, Imperial College London, Hammersmith Hospital Campus, London, United Kingdom; c Department of Surgery and Cancer, Faculty of Medicine, Imperial College London, St. Mary’s Hospital Campus, London, United Kingdom; d MRC Centre for Molecular Bacteriology and Infection, Department of Life Sciences, Faculty of Natural Sciences, Imperial College London, South Kensington Campus, London, United Kingdom; e Department of Biochemistry, Faculty of Medicine, Khon Kaen University, Khon Kaen, Thailand; f Department of General, Visceral, Transplant, Vascular, and Pediatric Surgery, University Hospital Wuerzburg, Wuerzburg, Germany; University of California, Irvine

**Keywords:** fecal microbiota transplantation, antibiotic effect, gut microbiota, metabolomics, weight loss surgery

## Abstract

Roux-en-Y gastric bypass (RYGB) is an effective weight loss surgery, resulting in a characteristic increase of fecal *Gammaproteobacteria*. The contribution of this compositional change to metabolic benefits of RYGB is currently debatable. Therefore, this study employed 16S rRNA gene sequencing and metabolic profiling to monitor the dynamic colonization of the RYGB microbial consortium and their metabolic impact on the host. Eleven Wistar rats received vancomycin and enrofloxacin, followed by fecal microbiota transplantation (FMT) of cecal slurry obtained from either RYGB- or sham-operated rats. Urine and feces from the microbiota recipients (RYGB microbiota recipients [RYGBr], *n* = 6; sham microbiota recipients [SHAMr], *n* = 5) were collected pre- and post-antibiotics and 1, 3, 6, 9, and 16 days post-FMT. No significant differences in body weight and food intake were observed between RYGBr and SHAMr. While neither group reached the community richness of that of their donors, by day 6, both groups reached the richness and diversity of that prior to antibiotic treatment. However, the typical signature of RYGB microbiome—increased *Enterobacteriaceae*—was not replicated in these recipients after two consecutive FMT, suggesting that the environmental changes induced by the anatomical rearrangements of RYGB could be key for sustaining such a consortium. The transplanted bacteria did not induce the same metabolic signature of urine and feces as those previously reported in RYGB-operated rats. Future work is required to explore environmental factors that shape the RYGB microbiota in order to further investigate the metabolic functions of the RYGB microbiota, thereby teasing out the mechanisms of the RYGB surgery.

**IMPORTANCE** Roux-en-Y gastric bypass (RYGB) surgery results in a long-term gut bacterial shift toward *Gammaproteobacteria* in both patients and rodents. The contribution of this compositional shift, or the RYGB bacterial consortium, to the metabolic benefit of the surgery remains debatable. It is unclear how well these bacteria colonize in an anatomically normal gut. This is a fundamental question in both defining the function of the RYGB microbiota and evaluating its potential as a nonsurgical treatment for obesity. We monitored the dynamic colonization of the RYGB bacterial consortium and observed that while approximately one-third of the bacterial taxa from the RYGB donor colonized in the gut of the nonoperated recipients, *Gammaproteobacteria* were unable to colonize for longer than 3 days. The study highlighted that a successful long-term colonization of *Gammaproteobacteria*-rich RYGB microbiota in nonsurgical animals requires key environmental factors that may be dictated by the intestinal anatomical modification by the surgery itself.

## INTRODUCTION

Originally developed as a surgical intervention for obesity, Roux-en-Y gastric bypass (RYGB) is now widely utilized as a highly effective form of metabolic surgery. In addition to producing long-term and sustained weight loss, it has been demonstrated to ameliorate the presence of type II diabetes mellitus (TIIDM) in over 80% of patients, thus making it far superior to current pharmacological interventions alone (1). Moreover, these beneficial metabolic alterations occur prior to and independent of weight loss (2, 3). However, the mechanisms underlying the profound effect of RYGB on energy homeostasis are incompletely understood.

RYGB is a multimodal surgical procedure resulting in a profound rearrangement of the gastrointestinal tract (4). The principal biological impact of these anatomical alterations has been characterized via the so-called B.R.A.V.E. effects: bile flow alteration, restriction of stomach size, altered flow of nutrients, vagal manipulation, and enteric gut and adipose hormone modulation (4). These biological alterations produce pleiotropic effects throughout most major organ systems that, in addition to the gastrointestinal system, include the brain, liver, pancreas, and adipose and muscle tissue (5). Furthermore, RYGB induces a unique alteration of the microbial community structure, characterized by a substantial increase in the proportion of *Gammaproteobacteria*, predominantly that of the *Enterobacteriaceae* family (4, 6). The relative abundance of species known to be associated with decreased adiposity, such as Akkermansia muciniphila, *Alistipes*, and Klebsiella pneumoniae, were augmented following bypass surgery (7–11). It was reported that following fecal microbial transplantation (FMT), the RYGB microbiota resulted in a reduction in adiposity, highlighting the potential transmissibility of adiposity through the gut microbiota (7). Another study demonstrated that germfree (GF) mice receiving RYGB microbiota showed lower postprandial peak glucose levels compared to mice that received sham microbiota (12, 13). However, these studies did not vigorously assess the similarity of the gut microbial composition between the donors and the recipients, which hinders the identification of the adiposity reduction-associated functional microbiota within the RYGB microbial consortium. Furthermore, recent publications suggested that some of the specific gut microbial communities associated with RYGB, e.g., A. muciniphila, may not be required to achieve the beneficial surgical outcomes (13, 14). Despite the conflicting evidence, it is crucial to investigate how well the RYGB microbial consortium colonizes in an anatomically normal gastrointestinal environment following FMT before studying the impact of this specific consortium on the host metabolism independent of the RYGB surgery. Previous studies utilizing GF models are limited in that prior to transplantation the animals exhibit immature gastrointestinal and immune functions. Consequentially, while colonization of a commensal microbiota appears successful, it can trigger a substantial immune activation and maturation of the gastrointestinal tract, which does not occur in antibiotic-treated conventionally raised animals.

RYGB surgery has been reported to alter host-microbe cometabolites in both rodents and humans, reflected by increased urinary concentrations of phenylacetylglycine/phenylacetylglutamine, indoxyl sulfate, and 4-cresyl glucuronide (4, 15). These metabolic changes could be attributed to the gut microbial shift toward the high abundance of *Enterobacteriaceae*. However, it is unknown to what extent the gut microbial changes contribute to the postsurgical host-microbe cometabolism, in comparison to those induced by the surgery itself. Therefore, the current study aimed to monitor the dynamic colonization of RYGB microbiota in a nonsurgical and broad-spectrum antibiotic-treated Wistar rat model and to investigate the impact of the colonized gut microbiota on the host metabolism.

## RESULTS

### Phenotypic differences between RYGBr and SHAMr following FMT.

**FIG 1:**
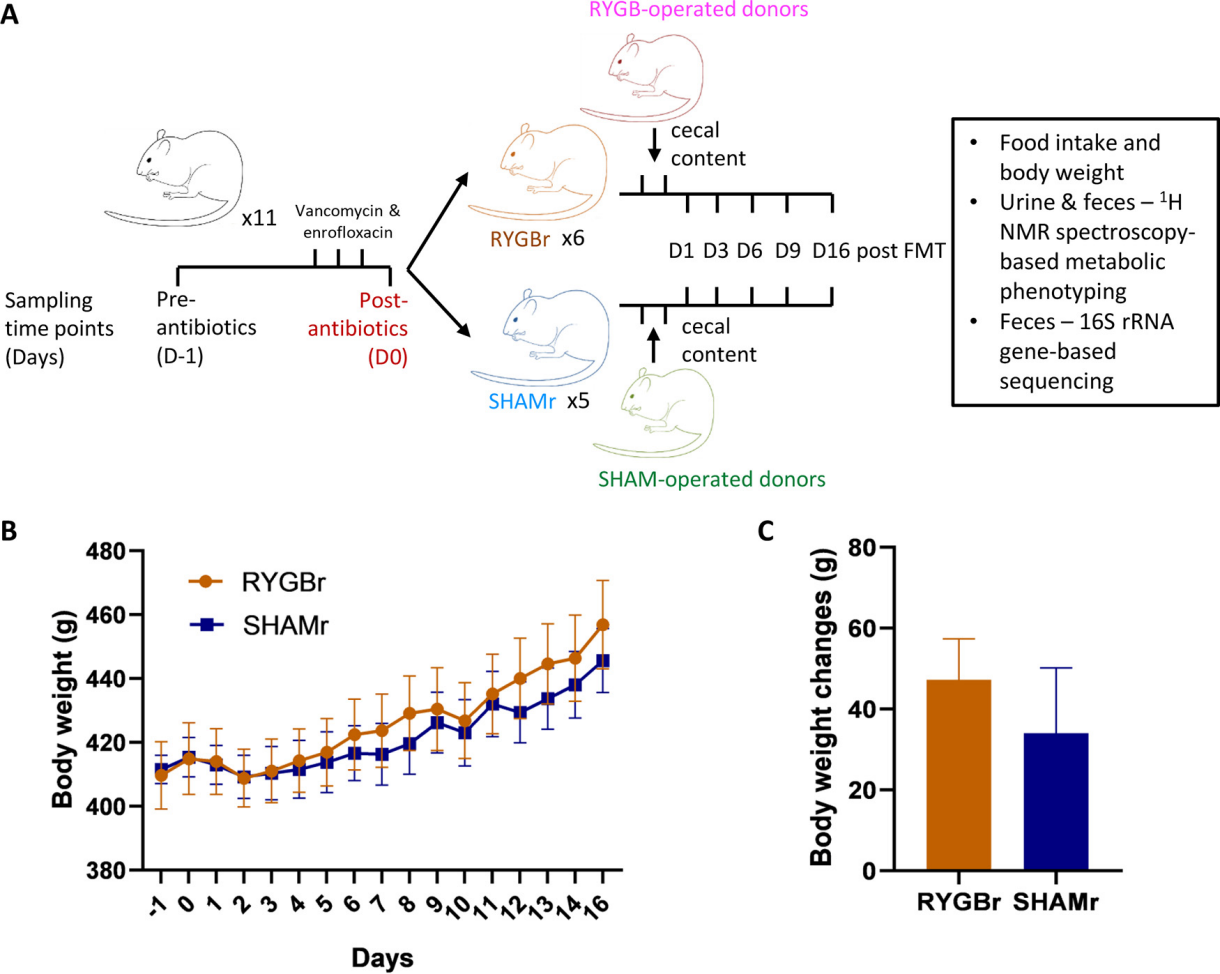
(A) Experimental design. (B) Body weight of the rats receiving either RYGBr (orange) or SHAMr (blue) microbial consortium over the 16-day period. (C) Weight changes between D-1 and D16 in RYGBr and SHAMr groups. Data are presented as means ± standard errors of the means (SEM) (error bars). FMT, fecal microbiota transplant; NMR, nuclear magnetic resonance; RYGBr, RYGB microbiota recipients; SHAMr, sham microbiota recipients.

The experimental design is illustrated in Fig. 1A. There was no significant difference in body weight or body weight changes between the RYGB microbiota recipients (RYGBr) and sham microbiota recipients (SHAMr) over the experimental duration (Fig. 1B and C). No significant differences were observed in food consumption between the two groups.

### Cecal bacterial composition of donors and differences of bacterial communities between donors and recipients at the baseline.

**FIG 2:**
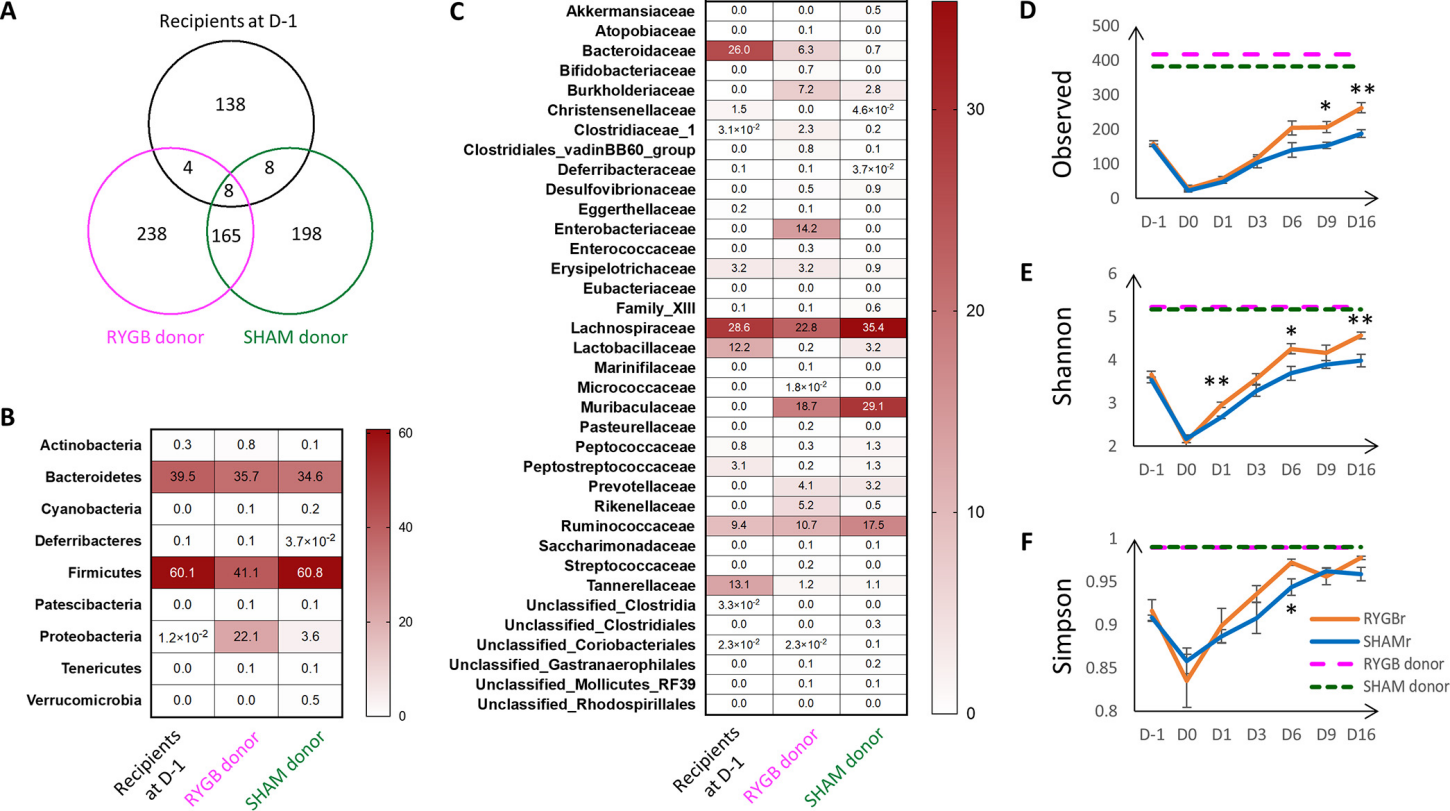
(A) Venn diagram of the number of taxa in RYGB donor, sham microbiota donor, and recipients at preantibiotics time point (D-1). (B and C) Relative abundances of bacteria at the phylum (B) and family (C) levels in RYGB and SHAM donors and the recipients at D-1. (E to G) Observed number of amplicon sequence variant (ASV) (E) and Shannon’s (F) and Simpson’s (G) diversity indices of the fecal samples at pre- and postantibiotics, and post FMT of RYGB or sham microbiota. Data are presented as means ± SEM. Significance between the RYGBr and SHAMr at each time point was determined using *t* test (normally distributed) or Wilcoxon test (not normally distributed). *, *P* < 0.05; **, *P* < 0.01.

A total of 415, 379, and 158 amplicon sequence variants (ASVs) were identified in the RYGB donor, sham microbiota donor, and recipients at the baseline (preantibiotic treatment, day before treatment [D-1]), respectively. A total of 238 out of 415 ASVs, 198 out of 379 ASVs, and 138 out of 158 ASVs were found to be unique to the RYGB donor, sham microbiota donor, and recipients at the baseline, respectively (Fig. 2A). *Firmicutes* and *Bacteroidetes* were dominant phyla in both donors and recipients; however, the abundance of *Proteobacteria* was much higher in the RYGB donor (22.1%) compared to the sham microbiota donor (3.6%) and recipients (0.012%) (Fig. 2B). The relative abundance of the *Enterobacteriaceae* family, which typifies the RYGB surgery-induced bacterial signature, was 14.2%, and it is the main contributor to the increased abundance of *Proteobacteria* phylum in the RYGB donor (Fig. 2C).

### Colonization of the donor bacteria in the recipients.

Antibiotics were given to the recipient animals prior to the microbial transplantation (Fig. 1A) and the antibiotic-induced metabolic and bacterial changes are described in supplemental material (see Fig. S1 and S2 and Table S1 in the supplemental material).

10.1128/mSystems.01047-20.1 FIG S1 (A) PCoA score plot of the fecal bacterial profiles from the recipients at preantibiotics and postantibiotics, PERMANOVA *P* value < 0.001 and *R*^2^ = 0.82. The percentage on each axis represents the amount of variation explained by the component. (B) The significantly changed bacterial families following antibiotic treatment. The data are presented as ln medians ± SEM. Two-way analysis of variance (ANOVA) was used to calculate the significance between the two time points, and Bonferroni’s multiple-comparison test was applied to correct *P* values. *, 0.01 < *P* < 0.05; ****, *P* < 0.0001. Following the treatment of antibiotics, a clear separation between pre- and post-antibiotics was observed in the principal-coordinate analysis (PCoA) scores plot (A). While ANCOM did not show any significant differences in ASVs between the two time points, we observed 7 bacterial families that were significantly altered by the antibiotic treatment by comparing the sum of the ASV reads at the family levels at pre-antibiotics with that at post-antibiotics. Fecal *Bacteroidaceae* (mainly *Bacteroides uniformis*), *Lachnospiraceae* (*Lachnospiraceae*_NK4A136_group, Unclassified_*Lachnospiraceae*), *Ruminococcaceae* (*Ruminococcaceae*_UCG-014 and Unclassified_*Ruminococcaceae*), and *Tannerellaceae* (mainly *Parabacteroides goldsteinii*) were found to be reduced, whereas *Erysipelotrichaceae* (mainly Unclassified_*Erysipelatoclostridium*), *Lactobacillaceae* (mainly unclassified *Lactobacillus*), and *Peptostreptococcaceae* (mainly *Romboutsia ilealis*) were increased following antibiotic treatment (B). Download FIG S1, TIF file, 0.7 MB. Copyright © 2020 Liu et al. 2020 Liu et al. This content is distributed under the terms of the Creative Commons Attribution 4.0 International license.

10.1128/mSystems.01047-20.2 FIG S2 OPLS-DA analysis of ^1^H NMR spectral data of fecal water (A and B) and urine (C and D) samples obtained preantibiotics (blue squares) and postantibiotics (red dots). Panels A and C are sevenfold cross-validated scores plots, indicating the group separation. Panels B and D are corresponding loading plot, indicating the metabolic changes contributing to the group separation. Peaks pointing upwards represent higher relative levels of metabolites at postantibiotics compared to pretreatment and vice versa. The colors of the peaks represent the square of correlation coefficient (*r*^2^) between the metabolite levels and group classification (i.e., pre- versus postantibiotics). The red peaks are highly correlated with the treatment, whereas the blue ones do not correlate with the treatment. The OPLS-DA model of feces showed *R*^2^*X* = 0.76, *R*^2^*Y* = 0.99, *Q*^2^ = 0.99, *P* = 0.005, where *R*^2^*X* represents the percentage of variation in the NMR spectral data explained by one PLS component and one orthogonal component, *R*^2^*Y* represents the percentage of variation in the class matrix (i.e., pre- versus postantibiotics) explained by one PLS component and one orthogonal component, *Q*^2^ represents the predictivity of the model, and the *P* value, calculated from the permutation test (200 permutations), indicates the significance of the model (cutoff = 0.05). The parameters from the OPLS-DA model of urine are *R*^2^*X* = 0.83, *R*^2^*Y* = 0.97, *Q*^2^ = 0.93, *P* = 0.005. Metabolite keys: 1, acetate; 2, acetoin; 3, alanine; 4, allantoin; 5, alpha-glucose; 6, aspartate; 7, beta-glucose; 8, butyrate; 9, choline; 10, *cis*-aconitate; 11, citrate; 12, tyrosine; 13, creatinine; 14, dimethylamine; 15, dimethylglycine; 16, formate; 17, fumarate; 18, glutamate; 19, glutarate; 20, glycine; 21, uracil; 22, guanidoacetate; 23, hippurate; 24, valine; 25, xylose; 26, hypoxanthine; 27, isoleucine; 28, lactate; 29, leucine; 30, lysine; 31, methionine; 32, methylamine; 33, nicotinate; 34, 2-oxoglutarate; 35, phenylacetylglycine; 36, phenylalanine; 37, 3-indoxylsulfate; 38, proline; 39, propionate; 40, pyruvate; 41, succinate; 42, taurine; 43, threonine; 44, 3-phenylpropionate; 45, TMAO (trimethylamine-*N*-oxide); 46, *trans*-aconitate; 47, trigonelline; 48, tryptophan. The antibiotic cocktail also induced a range of significant changes in urinary and fecal metabolite profiles. Orthogonal partial least squares-discriminant analysis (OPLS-DA) of the ^1^H NMR spectral data showed clear discrimination between pre- and post-antibiotic treatment (A and C). Whilst the effects appeared more pronounced in the feces, both the models were significant with a permutation *P* value of 0.005 at 200 permutes. A total of 16 and 20 discriminatory metabolites in feces and urine, respectively, were identified (Table S1). Fecal concentrations of acetate, butyrate, 3-phenylpropionate, acetoin, and several amino acids (i.e., alanine, valine, isoleucine, leucine, and methionine) were found decreased following antibiotic administration, while α-glucose, lactate, fumarate, pyruvate, choline, formate, and glycine were increased (B). Urinary concentrations of acetoin, acetate, glutarate, hippurate, valine, isoleucine, methylamine, phenylacetylglycine, 3-indoxylsulfate, pyruvate, succinate, 3-phenylpropionate, trimethylamine *N*-oxide (TMAO), and trans-aconitate were reduced, and these were accompanied by increases in the concentrations of cis-aconitate, creatinine, formate, glycine, guanidoacetate, and trigonelline (D). Download FIG S2, TIF file, 0.3 MB. Copyright © 2020 Liu et al. 2020 Liu et al. This content is distributed under the terms of the Creative Commons Attribution 4.0 International license.

10.1128/mSystems.01047-20.5 TABLE S1 Summary of fecal and urinary metabolite changes postantibiotic treatment. Key: F, feces; U, urine; s, singlet; d, doublets; dd, double of doublets; m, multiplets; t, triplets; q, quartets. ↓ and ↑ indicate decreased and increased relative levels of the metabolites postantibiotics compared to pretreatment, respectively. *r* represents the correlation coefficient between the metabolite levels and time points. Download Table S1, DOCX file, 0.02 MB. Copyright © 2020 Liu et al. 2020 Liu et al. This content is distributed under the terms of the Creative Commons Attribution 4.0 International license.

Following the transplantation of RYGB or sham microbial consortium, neither group of recipients reached the species richness or alpha diversity indices of their donors (Fig. 2D to F). However, bacterial species richness was restored to that of preantibiotic (D-1) levels by day 6 posttransplant in both RYGBr and SHAMr groups, and by day 9, species richness had surpassed that of their indigenous communities, with RYGBr exhibiting a significantly higher species richness compared to their SHAMr counterparts (Fig. 2D). Shannon’s diversity index of RYGBr consistently exceeded that of the SHAMr posttransplant (Fig. 2E), and a similar trend was observed in Simpson’s diversity index (Fig. 2F).

The number of ASVs transferred from the donors to the corresponding recipients are summarized in Table 1. The number of transferred ASVs from the donors increased from day 1 (D1) to day 16 (D16), whereas the number of ASVs that remained from the indigenous communities of the recipients at the baseline (D-1) were relatively stable. While a higher number of ASVs from the RYGB donor were transferred to the recipients compared to that from the sham microbiota donor at all time points, only 31.8% of ASVs from the RYGB donor (132 out of 415) and 10.8% (41 out of 379) from the sham microbiota donor were transferred to their corresponding recipients at D16. At the family level, 50% of ASVs in *Rikenellaceae* (3 out of 6), 13.8% in *Ruminococcaceae* (15 out of 109), 2.7% in *Lachnospiraceae* (4 out of 150), and 6.4% in *Muribaculaceae* (3 out of 47) were transferred from the sham microbiota donor to SHAMr at D16 (Table S2). In contrast, 72.7% of ASVs belonging to *Bacteroidaceae* (8 out of 11), 75% in *Desulfovibrionaceae* (3 out of 4), 61.5% in *Rikenellaceae* (8 out of 13), 38.9% in *Ruminococcaceae* (35 out of 90), 28.8% in *Lachnospiraceae* (42 out of 146), and 18.5% in *Muribaculaceae* (10 out of 54) were transferred from the RYGB donor to the RYGBr at D16 (Table S2). However, *Enterobacteriaceae*, the bacterial family that increased significantly post-RYGB surgery, was not able to colonize after day 3 posttransplantation, evidenced by 4 and 1 out of 13 ASVs from the RYGB donor being present in the RYGBr at D1 and D3 posttransplantation and none from D6 to the end of the experiment (Table S2). In contrast to the colonization of the bacteria from the donor in the recipients, a smaller number of ASVs from the indigenous communities of the recipients remained posttransplantation (Table 1). A total of 81 ASVs from the *Lachnospiraceae* family were exclusively present in the recipients at the baseline, and at D16, only 14 and 9 out of them remained in RYGBr and SHAMr, respectively (Table S2). There were 29 ASVs from *Ruminococcaceae* in the recipients at the baseline, 2 and 3 out of which remained in RYGBr and SHAMr, respectively, at D16 (Table S2).

**TABLE 1:** Amplicon sequence variants transferred from RYGB or SHAM donors to their corresponding recipients^*a*^

Time point	Number of ASVs					
	From RYGB donor only	Remained from the RYGBr’s indigenous at baseline (D-1) only	From both RYGB donor and indigenous communities of the recipients	From SHAM donor only	Remained from the SHAMr’s indigenous communities at baseline (D-1) only	From both SHAM donor and indigenous communities of the recipients
D1	26	8	4	10	10	4
D3	41	13	4	18	16	4
D6	77	29	7	23	19	3
D9	86	24	8	32	22	5
D16	132	26	8	41	21	6

a Colonization of a taxon from the donor in ≥50% recipient rats was counted as a transferred taxon.

10.1128/mSystems.01047-20.6 TABLE S2 Summary of the number of amplicon sequence variants (ASVs) in each bacterial family that were transferred from donors to the corresponding recipients or remained in the recipients from the baseline (preantibiotics [D-1]). Download Table S2, DOCX file, 0.03 MB. Copyright © 2020 Liu et al. 2020 Liu et al. This content is distributed under the terms of the Creative Commons Attribution 4.0 International license.

**FIG 3:**
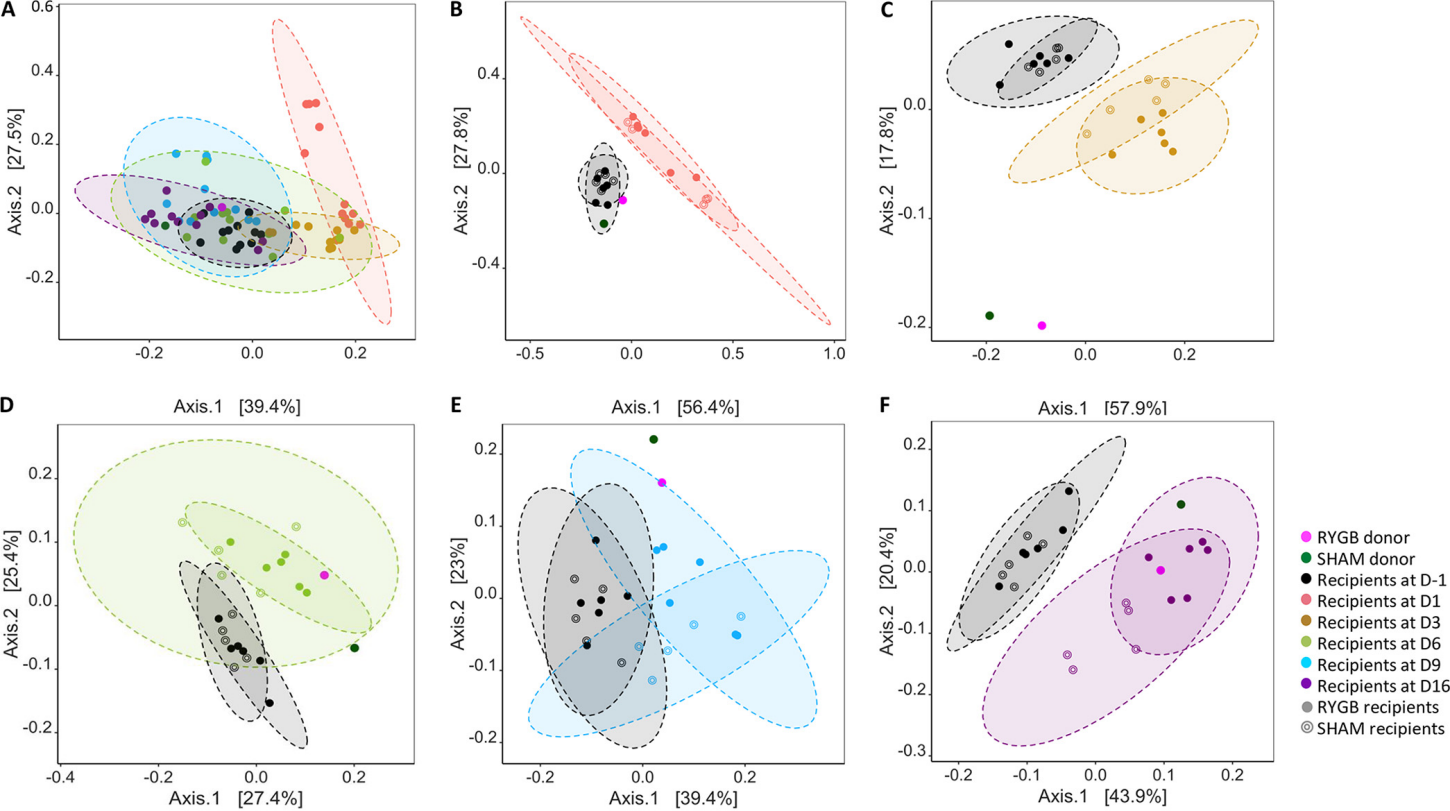
PCoA scores plot of weighted UniFrac fecal bacterial profiles of RYGB donor (pink), sham microbiota donor (dark green), microbiome recipients at the taxa level at preantibiotics (D-1, black) and 1 (salmon), 3 (yellow), 6 (light green), 9 (blue), and 16 (purple) days after microbiota transplantation (A), PERMANOVA *P* < 0.001 and *R*^2^ = 0.51. PCoA score plots of weighted UniFrac fecal bacterial profiles of RYGB and sham microbiota donors, their corresponding recipients at D-1 together with each of the posttransplantation time points (D1 [B], D3 [C], D6 [D], D9 [E], and D16 [F]). PERMANOVA *P* value < 0.001 between any two compared time points in the recipients. PERMANOVA *R*^2^ for panels B to F were 0.51, 0.57, 0.30, 0.42, and 0.44, respectively. The percentage on each axis represents the amount of variation explained by the component.

To visualize the bacterial colonization in the recipients over a 16-day period, weighted UniFrac principal-coordinate analysis (PCoA) was conducted based on the ASV profiles from RYGB and sham microbiota donors, and microbial recipients at D-1, D1, D3, D6, D9, and D16. A time-related shift was observed with D1, and D3 deviated from the cluster of the remaining time points (Fig. 3A; Fig. S3). At each of the posttransplantation time points, both RYGBr and SHAMr were separated from their corresponding baseline profiles (Fig. 3B to F) and gradually moved closer to the donor profiles by D16 (Fig. 3F). PERMANOVA (permutational analysis of variance) showed a *P* value of <0.001 for the comparison of all time points (Fig. 3A) and between the two compared time points (Fig. 3B to F). Furthermore, a separation between RYGBr and SHAMr was also observed from D3 onwards (Fig. 3C to F). These observations are consistent with the aforementioned data that a higher number of ASVs from the RYGB donor were transferred to RYGBr compared to the sham microbiota donor-recipient pair, suggesting that a larger fraction of the taxa from the RYGB donor could colonize in the recipients.

10.1128/mSystems.01047-20.3 FIG S3 PCoA score plot of fecal bacterial profiles of RYGB donor (pink), SHAM donor (dark green), microbiome recipients at the taxon level at preantibiotics (D-1, black), postantibiotics (D0, dark red) and 1 (salmon), 3 (yellow), 6 (light green), 9 (blue), and 16 (purple) days after microbiota transplantation. PERMANOVA *P* value < 0.001 and *R*^2^ = 0.67. The percentage on each axis represents the amount of variation explained by the component. Download FIG S3, TIF file, 1.1 MB. Copyright © 2020 Liu et al. 2020 Liu et al. This content is distributed under the terms of the Creative Commons Attribution 4.0 International license.

**FIG 4:**
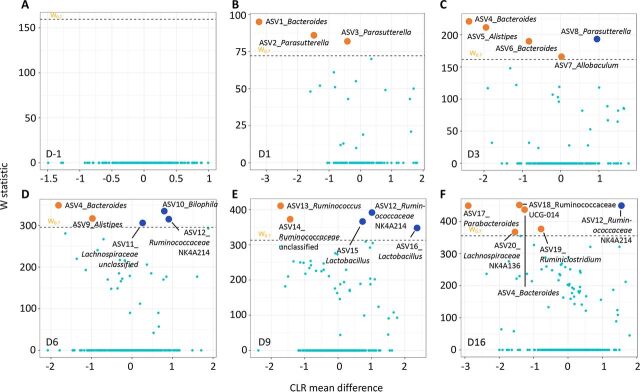
Taxonomy differential analysis on ASVs between the RYGBr and SHAMr groups at D-1 (A), D1 (B), D3 (C), D6 (D), D9 (E), and D16 (F) using ANCOM analysis. The *x* axis shows the centered log ratio (CLR) mean difference, and the *y* axis shows the W statistic representing the strength of ANCOM test, where a cutoff value of 0.7 was used. Sky blue dots represent nonsignificant ASVs between the two groups, whereas orange or dark blue dots represent ASVs that are significantly higher in the RYGBr or SHAMr group compared to the other group. The dots are labeled with ASV ID_genus. The sequences and taxonomy of these ASVs are summarized in Table S3 in the supplemental material.

**FIG 5:**
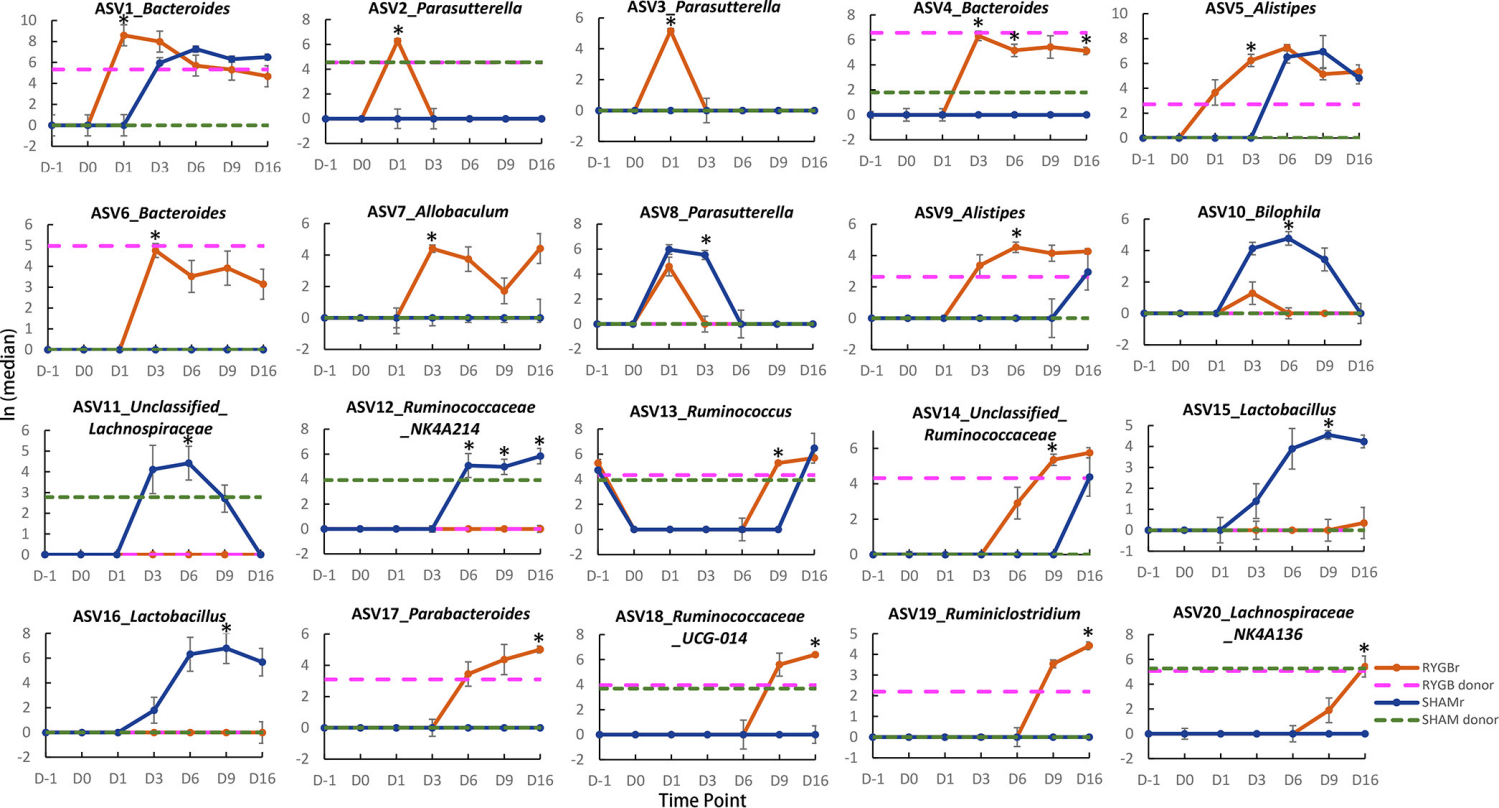
Natural log values of ASV reads from the RYGB donor (pink dashed line) and SHAM donor (green dashed line) and the RYGBr (orange) and SHAMr (dark blue) at D-1 (preantibiotics), D0 (postantibiotics), and D1, D3, D6, D9, and D16 postmicrobial transplantation. These 20 ASVs were found to be significantly different between RYGBr and SHAMr at one or more time points postmicrobial transplantation based on ANCOM analysis. The data are presented as ln medians ± standard errors of the means (SEM) (error bars). An asterisk represents a significant difference between RYGBr and SHAMr at that time point based on ANCOM analysis. The detailed taxonomy and sequences of these ASVs are summarized in Table S3.

Analysis of composition of microbiomes (ANCOM) analysis was subsequently used to investigate significantly different ASVs between RYGBr and SHAMr at each time point. There was no significant difference between the two groups prior to the microbial transplantation (Fig. 4A). A total of 20 ASVs were found to be significantly different between RYGBr and SHAMr posttransplantation (Fig. 4B to F; see also Table S3). ASVs belonging to the bacterial genera of *Bacteroides*, *Parasutterella*, *Alistipes*, *Allobaculum*, *Ruminococcus*, *Ruminiclostridium*, and *Parabacteroides* were found to be higher in RYGBr compared to SHAMr, whereas *Bilophila*, *Ruminococcaceae* NK4A214 group, *Lactobacillus*, and an unclassified *Lachnospiraceae* group were higher in SHAMr. Across the posttransplantation time points, *Bacteroides* (ASV4 and ASV6) from the RYGB donor started to colonize in the gut of RYGBr from D3, whereas *Parabacteroides* (ASV17) and *Ruminiclostridium* (ASV19) started from D6 and D9, respectively (Fig. 5). *Ruminococcaceae* NK4A214 group (ASV12) was present only in the sham microbiota donor and started to colonize in SHAMr from D6 (Fig. 5). While some bacteria, such as *Bacteroides* (ASV1), *Alistipes* (ASV5 and ASV9), and unclassified *Ruminococcaceae* (ASV14) were detected only in the RYGB donor, they were able to colonize in both RYGBr and SHAMr (Fig. 5). In contrast, *Ruminococcaceae*_UCG-014 group (ASV18) and *Lachnospiraceae*_NK4A136 group (ASV20) were present in both the RYGB and sham microbiota donors, but they colonized only in RYGBr from D9 but not in SHAMr, suggesting a favored gastrointestinal environment in the RYGBr for colonization of these bacteria. Of note, no significant differences in the ASVs belonging to *Enterobacteriaceae*, the key signature post-RYGB surgery, was observed between RYGBr and SHAMr.

10.1128/mSystems.01047-20.7 TABLE S3 Summary of significantly different amplicon sequence variants (ASVs) between RYGBr and SHAMr group at each time point based on ANCOM analysis with a cutoff W statistic of 0.7. “↑” or “↓” means a higher or lower relative abundance in RYGBr compared to SHAMr. ASV IDs correspond to those shown in Fig. 4. p, phylum; c, class; o, order; f, family; g, genus. Download Table S3, DOCX file, 0.02 MB. Copyright © 2020 Liu et al. 2020 Liu et al. This content is distributed under the terms of the Creative Commons Attribution 4.0 International license.

10.1128/mSystems.01047-20.10 TABLE S6 List of bacterial families and fecal and urine metabolites included in the DIABLO correlation analysis. Download Table S6, DOCX file, 0.01 MB. Copyright © 2020 Liu et al. 2020 Liu et al. This content is distributed under the terms of the Creative Commons Attribution 4.0 International license.

### Urinary and fecal metabolic differences between RYGBr and SHAMr posttransplantation.

Urinary metabolic profiles were not significantly different between RYGBr and SHAMr at any time point. Fecal metabolic profiles on day 3, but not the other time points, showed significant differences between RYGBr and SHAMr, which were contributed by higher relative levels of amino acids, such as leucine, isoleucine, valine, tyrosine, and phenylalanine in the SHAMr compared to RYGBr (Fig. S4).

10.1128/mSystems.01047-20.4 FIG S4 Loading plot of OPLS-DA comparison between RYGBr and SHAMr metabolic profiles of feces at 3 days after microbial transplantation. *R*^2^*X* = 0.34, *R*^2^*Y* = 0.97, *Q*^2^ = 0.74, permutation *P* = 0.005. Key: Phe, phenylalanine; Tyr, tyrosine; Val, valine; Leu, leucine; Ile, isoleucine. Download FIG S4, TIF file, 1.0 MB. Copyright © 2020 Liu et al. 2020 Liu et al. This content is distributed under the terms of the Creative Commons Attribution 4.0 International license.

### Integrative analysis of body weight, urinary and fecal metabolites, and bacteria.

**FIG 6:**
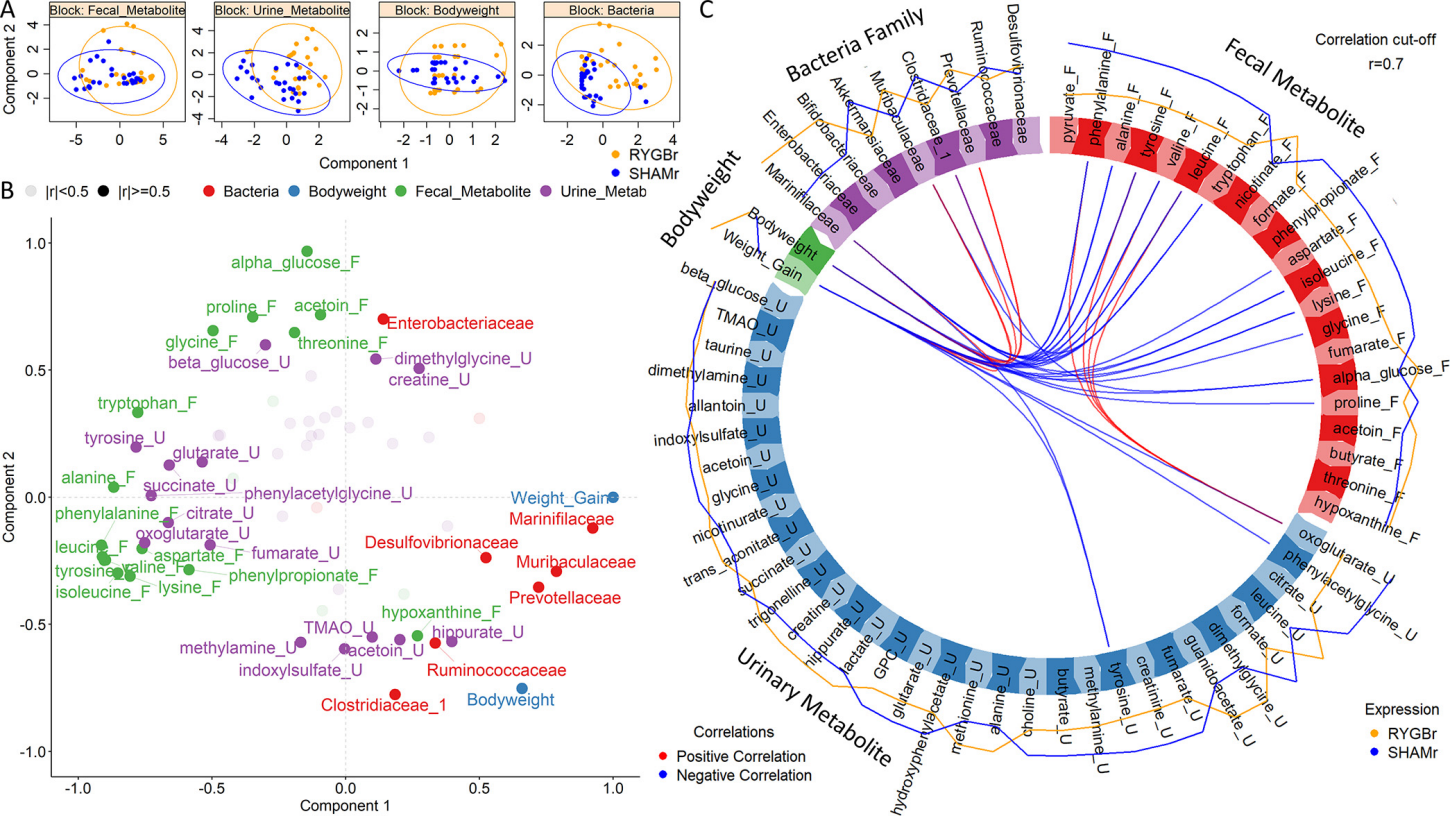
Integration of urinary metabolites (names ending with “_U”), fecal metabolites (names ending with “_F”), fecal bacteria at familial level, body weight, and weight gain using a DIABLO algorithm in the mixOmics R package. Data from six time points (preantibiotics, days 1, 3, 6, 9 and 16) were included for correlation analysis. (A) Component plots for each data set depicting the clustering of subjects with respect to RYGBr (orange) and SHAMr (blue) group. (B) Variables plots generated by calculating the correlation between variables. Variables, which are strongly positively correlated, are projected closely to each other; the longer the distance between the variables and the origin, the stronger the correlation between the variables. Correlations above the threshold (*r* = 0.5) were labeled in the plot. (C) Circos plot depicting correlations between variables in different data sets. Red and blue lines inside the circle indicate positive and negative correlation between the variables, respectively. Correlations above the threshold (*r* = 0.7) are depicted. The lines around the ideogram indicate average levels of expression of each variable from RYGBr (orange) and SHAMr (blue) groups. The further the line to the circle, the higher expression the variable is compared to the other group.

To determine whether there are any differences between RYGBr and SHAMr groups across different data sets, an integrative approach was used to model fecal and urinary metabolic profiles, body weight and body weight changes, and bacteria at the family level. RYGBr and SHAMr could be discriminated along the first component of the model based on bacterial profiles, while the differences were not apparent in the metabolic data sets and body weight (Fig. 6A). *Marinifilaceae*, *Muribaculaceae*, *Prevotellaceae*, and *Desulfovibrionaceae* were highly correlated with the classes and had the highest contribution to the model (Fig. 6B). *Prevotellaceae*, *Clostridiaceae*_1, and *Muribaculaceae* were highly positively correlated with body weight, and *Marinifilaceae* was most correlated with weight gain. Urinary phenylacetylglycine (PAG), 2-oxoglutarate and tyrosine and fecal amino acids had a strong negative correlation with body weight or weight gain. In contrast, fewer correlations between the bacteria and metabolites were observed (Fig. 6C).

## DISCUSSION

In this study, we assessed the dynamic colonization of the gut bacteria from RYGB- or sham-operated rats in a nonsurgical and antibiotic-treated Wistar rat model and monitored the metabolic disturbances in urine and feces caused by these colonized bacteria. We observed that approximately one-third of the ASVs from the RYGB donor was transferred to RYGBr, whereas only one-tenth from the sham microbiota donor was transferred to SHAMr by the end of the experiment. These transferred bacteria did not result in weight loss of the animals or metabolic alterations in urine and feces as those (e.g., host-microbe cometabolites) observed in the RYGB-operated patients and animals. These observations suggested that these colonized bacteria in RYGBr may not be responsible for the metabolic changes in host-microbe metabolism following RYGB surgery and the metabolic benefit of reduced body weight.

Following FMT, the Shannon diversity index of RYGBr becomes very similar to that of their donor, yet neither the RYGBr nor SHAMr community reached their donor richness. This observation could result from multiple factors. First, the donor samples were from the cecum, which is a typical sample type used in FMT in animal models. The cecal samples normally exhibit higher numbers of the microbiota compared to feces (16). Second, the donors were from an animal facility in Germany, whereas the microbial recipients were from the United Kingdom. Animals from different facilities may have different bacterial richness. Third, the higher alpha diversity could be due to the RYGB surgery as previously reported (17, 18). In our study, we also noted a higher number of observed ASVs in the RYGB donor compared to the sham microbiota donor. Compared to the indigenous microbial community of the recipients, by day 6 post-FMT, both RYGBr and SHAMr reached the richness and diversity of their microbiota prior to antibiotics, suggesting that any suppressive effects from antibiotic treatment did not hamper the successful colonization of many of the transplanted communities. A recent study showed that treatment with or without antibiotics did not change the overall microbial dissimilarity between the recipients and the donors, but antibiotics induced specific genus-level differences, such as improved colonization of *Bifidobacterium*, *Adlercreutzia*, *Enterorhabdus*, *Odoribacter*, and *Alkalibacterium* in the recipients (19).

In agreement with previous literature, the RYGB donor exhibited an increase in the relative abundance of *Gammaproteobacteria*, dominated by members of the family *Enterobacteriaceae* (4). However, this typical signature of the RYGB microbial consortium was not replicated in the nonsurgical rats based on our findings. Previous studies have used GF models to study the impact of the RYGB microbiota on the glucose metabolism and adiposity of the host (7, 11), and similar to our observations, the abundance of *Enterobacteriales* from *Gammaproteobacteria* in RYGB recipients dropped from 40% at 1 day posttransplantation to approximately 18% on day 2 and <5% on day 3 (7). Taken together, this finding indicates that the *Gammaproteobacteria*-rich microbiota is unique to the gut environment post-RYGB surgery, and such a bacterial composition is unlikely to be mimicked using simple microbial transplantation in either germfree or antibiotic-treated animals. RYGB surgery encompasses a profound rearrangement of the gastrointestinal tract, resulting in major changes to the gut luminal milieu. Such changes have been postulated to favor the growth of *Proteobacteria* and include an increased glucose absorption (20), alterations in gut luminal acidity (21), and an increase in dissolved oxygen (6). While the instrumental factor promoting the bloom in *Gammaproteobacteria* remains undetermined, these data suggest that the selective pressures resulting from surgery-induced environmental alterations act as a prerequisite for the increased presence and community stability of this bacterial class.

In contrast to the *Enterobacteriaceae*, the *Parabacteroides* genus from the *Porphyromonadaceae* family, *Alistipes* genus from *Rikenellaceae* family, *Ruminiclostridium* genus from *Ruminococcaceae* family, and *Bacteroides* genus from *Bacteroidaceae* family were present in the RYGB donor only and successfully colonized in RYGBr. These bacteria have been reported to be negatively associated with obesity. For example, *Parabacteroides* was found to be inversely correlated with lipid consumption in obese diabetic women who underwent RYGB surgery (22). Parabacteroides distasonis has been shown to reduce weight gain, hyperglycemia, and hepatic steatosis in a high-fat diet-fed *ob/ob* mouse model (23). Succinate production of P. distasonis activated the intestinal gluconeogenesis through succinate binding with fructose-1,6-bisphosphatase, whereas lithocholic acid and ursodeoxycholic acid production of P. distasonis activated the farnesoid X receptor (FXR) signaling pathway and reduced hyperlipidemia and improved gut barrier integrity (23). Another species from this genus, P. goldsteinii, was also shown to reduce obesity, enhance intestinal integrity, and reduce inflammation and insulin resistance (24). In our study, while *Parabacteroides* colonized in RYGBr, we did not observe a reduction in body weight, which could be due to a relatively low count in this genus and/or a relatively short follow-up period (from D6 to D16) since the colonization of this bacterial genus. *Alistipes* and *Ruminiclostridium* were enriched in the healthy pregnant women compared to women with gestational diabetes mellitus (GDM) (48). Furthermore, a study based on 1,914 Chinese adults showed that *Alistipes* and *Parabacteroides* are negatively associated with body mass index, waistline, and serum lipid levels (25). We also observed the growth of the *Bacteroides* genus (i.e., ASV4 and ASV6 [Bacteroides vulgatus]) in RYGBr but not in SHAMr, while Bacteroides thetaiotaomicron (ASV1) colonized in both RYGBr and SHAMr. A lower relative abundance of fecal B. vulgatus was reported in obese individuals compared to lean children (26). However, a conflicting finding showed that this bacterial species decreased in the obese children following a 16-week supplement of oligofructose-enriched inulin along with significant body weight and fat reduction (27). These evidences suggest that the colonization of these bacterial genera in RYGBr may be able to induce metabolic benefits in a long-term follow-up, which warrants further investigation.

Another observation was that *Prevotellaceae*, *Clostridiaceae*_1, and *Muribaculaceae* families were highly positively correlated with body weight and *Marinifilaceae* was most correlated with weight gain. However, *Prevotellaceae* and *Muribaculaceae* have been linked to the lean phenotype. Similar to *Bacteroides*, *Prevotella* from *Prevotellaceae* family was found to be highly abundant in children from rural African villages and mice fed a high-fiber diet (28). Furthermore, mung bean supplementation-induced reduction in obesity was proposed to be via promoting the relative abundance of *Muribaculaceae* in a mouse model (29). The fecal abundance of *Clostridiaceae* was found to be lower in American obese individuals compared to the participants with normal weight based on two independent study populations (30). However, consistent with our observation of a positive correlation between body weight and *Clostridiaceae*, a higher abundance of this bacterial family was observed in obese adolescents compared to the lean individuals (31). Moreover, the translocation of the gut microbiota to extraintestinal tissues has been reported to link to type 2 diabetes, and *Marinifilaceae* was found to be enriched in the mesenteric adipose tissue of individuals without diabetes in contrast to patients with diabetes (32), but its link to body weight is unclear. Thus, the causal relationship between these bacterial families and obesity needs further investigation.

To counter the limitations imposed through the use of GF models, conventionally raised rats that had been pretreated with antibiotics were utilized in this study. The purpose of using antibiotics prior to FMT was to eliminate a substantial proportion of the indigenous bacteria, thus reducing competition and consequentially enhancing the successful colonization of an exogenous consortium. Consistent with previous studies (33, 34), following antibiotic treatment we observed reductions in overall gut bacterial abundances and bacterial metabolic activities, such as choline metabolism, host-microbe cometabolites, short-chain fatty acid production, and protein degradation. In addition, we observed an increased abundance of *Lactobacillus* (a Gram-positive and facultative anaerobic genus of bacteria). This increase could be due to the presence of intrinsic antibiotic resistance genes in this genus, as an intrinsic resistance to vancomycin in some lactobacilli has been characterized (35). For example, the terminal d-alanine residue, which is a binding point with vancomycin, is replaced by d-lactate or d-serine in the muramyl pentapeptide and therefore prevents the vancomycin binding to the bacterial cells (35). While many species and strains from the *Lactobacillus* genus are used in food fermentation processes and probiotics, the presence of intrinsic antibiotic resistance genes in these bacteria and the possibility of horizontal gene transfer from these bacteria to pathogenic bacteria could pose some potential safety issues (35, 36).

### Conclusion.

We showed that a subset of the RYGB bacterial consortium was transferred to the antibiotic-treated nonoperated rats. However, the typical signature of the RYGB microbiome—the increased *Enterobacteriaceae*—was not replicated in these recipients after two consecutive FMTs. Our results suggest that this specific bacterial composition could be highly dependent upon the environmental alterations induced by the anatomical rearrangements of the RYGB surgery. The transplanted bacteria did not induce the same metabolic signature of urine and feces as those previously reported in RYGB-operated rats. Furthermore, there was no evidence of any long-term suppressive effects of antibiotic treatment in the colonization of the transplanted microbiota. Future work is required to explore the environmental factors that shape the RYGB microbiota in order to further investigate the metabolic functions of the RYGB microbiota, thereby teasing out the mechanisms of the RYGB surgery.

## MATERIALS AND METHODS

### Animal experiments.

All animal experiments were performed under a license issued by the Home Office UK (PPL 70/8078). Nonobese male Wistar rats (*n* = 11) were purchased from Charles River Laboratories and individually housed under a 12-h light (7 a.m.-7 p.m.), 12-h dark cycle (7 p.m.-7 a.m.). Standard chow and water were available *ad libitum*. After a 2-week acclimatization period, the rats (approximately 11 weeks old) were administered an antibiotic mixture of vancomycin (50 mg · kg^−1^· day^−1^) and enrofloxacin (50 mg · kg^−1^ · day^−1^) via oral gavage for 3 days. Twenty-four hours after the cessation of antibiotics, rats were randomly assigned RYGB microbiota recipients (RYGBr; *n* = 6) or sham microbiota recipients (SHAMr; *n* = 5). The RYGBr and SHAMr received their respective microbial transplant via oral gavage (1.5 ml of cecal slurry per dose, one per day for 2 days). Each donor sample was pooled from the cecal contents of high-fat diet-fed rats having undergone either Roux-en-Y gastric bypass (RYGB, *n* = 6) or sham surgery (*n* = 6) according to the technique previously described (4). Glycerol (10%) was added to donor cecal samples to preserve the bacteria before storing it at –80°C. Urine and feces were collected at 8:30 a.m. at preantibiotics (day before antibiotics [D-1]) and postantibiotics (day 0 [D0]), and 1, 3, 6, 9, and 16 days following microbial transplant (D1 to D16) (Fig. 1A). All biofluid samples were subjected to metabolic analysis using ^1^H nuclear magnetic resonance (NMR) spectroscopic analysis, and fecal samples were additionally subjected to bacterial profiling using 16S rRNA gene sequencing.

### Metabolic profiling of biofluids.

NMR buffer was prepared by dissolving 20.4 g KH_2_PO_4_ (Sigma-Aldrich) in 80 ml D_2_O. Additionally, 100 mg 3-(trimethylsilyl)propionic-2,2,3,3-d_4_ acid sodium salt (TSP; Sigma-Aldrich) and 13 mg sodium azide (NaN_3_; Sigma-Aldrich) were dissolved in 10 ml deuterium oxide (D_2_O; Sigma-Aldrich). The solutions were sonicated together giving a 1.5 M KH_2_PO_4_ solution by adding D_2_O to 100 ml, and the pH was adjusted to 7.4 via the addition of KOH pellets (Sigma-Aldrich). Prior to preparation, urine samples were thoroughly defrosted and vortexed for 5 s. Samples were then centrifuged at 14,000 × *g* for 10 min at 4°C. Subsequently, a 300-μl aliquot of the sample was combined with 240 μl D_2_O and 60 μl buffer. Each sample was then spun for 10 s, and 580-μl aliquot was transferred to a 5-mm-diameter NMR tube for ^1^H NMR spectrum acquisition. For each fecal sample, one pellet was weighed and homogenized with high-performance liquid chromatography (HPLC)-grade water at a ratio of 1 mg feces to 1 μl water. Samples were then sonicated at 25°C for 5 min and vortexed for 2 min before centrifugation at 14,000 × *g* and 4°C for 15 min, repeated three times. After the supernatant was transferred to a new Eppendorf tube, the same volume of water was added, and the process was repeated. The pooled supernatant obtained from two cycles (480 μl) was mixed with 60 μl D_2_O and 60 μl potassium phosphate buffer described previously. The sample was fast spun, and a 580-μl aliquot was transferred to a 5-mm-diameter NMR tube. Samples were randomized, and ^1^H NMR spectra for both biofluid samples were obtained via a Bruker 800-MHz spectrometer (Bruker; Rheinstetten, Germany) at an operating ^1^H frequency of 800 MHz and a constant temperature of 300 K. One-dimensional (1D) ^1^H NMR spectra data were obtained utilizing the NMR pulse sequence (recycle delay [RD]-90°-*t*_1_-90°-*t_m_*-90°-acquisition) where *t*_1_ (short delay between the two 90° pulses) = 4 μs, *t_m_* (mixing time) = 100 ms, and 90° pulse = ∼10 μs. Selective irradiation was utilized during a RD of 4 s and *t_m_*_._ Thirty-two scans were obtained and collected to form 65,536 data points for each sample and a spectral width of 15 ppm.

### Data preprocessing and statistical data analysis of metabolic profiles.

The ^1^H NMR spectra obtained for both fecal water and urine samples were imported into MATLAB (R2014a), phased, referenced, and baseline corrected, and the resultant 10 ppm spectra were digitized into 20K data points, resolution = 0.0005 (34). Signals corresponding to water (δ^1^H, 4.69 to 4.90), urea (δ^1^H, 5.60 to 6.02, only in urinary spectra) and TSP (δ^1^H, −1 to 0.60) were removed prior to recursive segment-wise peak alignment (37) and normalization using the probabilistic quotient method (38). Principal component analysis (PCA) and orthogonal projections to latent structures discriminant analysis (OPLS-DA) were constructed on the processed data following mean centering and unit variance scaling, performed using the statistical packages in MATLAB. For each pairwise comparison, one predictive component and one orthogonal component were used to obtain the model. Two hundred permutation tests were performed to validate the goodness of the OPLS-DA model, with a *P* value < 0.05 regarded as a valid model. Significantly altered peak intensities were calculated by univariate analysis with a *P* value of <0.05 after Benjamini-Hochberg correction. Metabolite identification was achieved by STOCSY (39), Chenomx Software (Alberta, Canada), and searching previous literature database (34, 40, 41).

### 16S rRNA gene sequencing of fecal bacteria.

16S rRNA gene sequencing was used to determine the bacterial composition of fecal samples using the Illumina MiSeq. DNA was extracted from 250 mg fecal pellets using the QIAamp PowerFecal DNA kit (Qiagen, Crawly, UK) according to the manufacturer’s instructions, with an additional bead-beating step using a bead beater to aid with the homogenization at 4500 rpm for 45 s. The extracted DNA was subsequently quantified using the Qubit 2.0 fluorometer and diluted to 5 ng/μl. Sample libraries were prepared following Illumina’s protocols with a few modifications (42). MiSeq amplicon PCR was utilized to amplify the V1-V2 region of the 16S rRNA (see Table S4 in the supplemental material). Sequencing was performed on an Illumina MiSeq instrument with the MiSeq reagent kit v3 (Illumina) using paired-end 300-bp chemistry.

10.1128/mSystems.01047-20.8 TABLE S4 16S V1-V2 MiSeq primers (parts in bold are adapter sequences). Download Table S4, DOCX file, 0.01 MB. Copyright © 2020 Liu et al. 2020 Liu et al. This content is distributed under the terms of the Creative Commons Attribution 4.0 International license.

### Data preprocessing and statistical data analysis of bacterial profiles.

The resulting sequences were further processed using R v.3.6.3 and RStudio v.1.2.5033 with DADA2 v.1.14.1 (43) package. The forward and reverse reads were trimmed with the quality score 30 as the cutoff. Taxonomic labeling of amplicon sequence variants (ASVs) was done with a naive Bayesian classifier and a SILVA v.128 training set (44). A total of 2131 ASVs were detected. The taxon filter and prevalence control cutoff was set at ASVs ≥ 4 counts in 6.5% of the total number of samples excluding the two donor samples. This filter was set to ensure that any ASV, which was present in a particular treatment at a given time point, was not excluded from the analysis. After the filter was applied, the resulting data set consisted of 1,033 ASVs. The number of reads per sample pre- and post-rarefaction are listed in Table S5. Further analysis was conducted using the package phyloseq v.1.30.0 (45). For alpha diversity, rarefaction was done with the minimum sequence depth of 16,639 counts, and then observed reads, Shannon index, and Simpson index were calculated. The comparisons of alpha diversity indices between RYGBr and SHAMr group were tested using a *t* test (normally distributed) or Wilcoxon test (not normally distributed). For beta diversity, a weighted UniFrac distance matrix was created and ordinated using principal-coordinate analysis (PCoA). PERMANOVA was analyzed using the vegan (46) package in R to compare the bacterial profiles at different time points. The differential analysis of ASVs was performed using analysis of composition of microbiomes (ANCOM) (47). The W statistic was calculated using ANCOM to represent the strength of the test, with 0.7 as the cutoff. These differential ASVs between RYGBr and SHAMr at any time point were selected, and the median abundances with natural log transformation of these ASVs in RYGBr and SHAMr at all time points were shown using line plots to visualize the time-dependent changes.

10.1128/mSystems.01047-20.9 TABLE S5 The number of reads per sample pre- and post-rarefaction. Download Table S5, DOCX file, 0.02 MB. Copyright © 2020 Liu et al. 2020 Liu et al. This content is distributed under the terms of the Creative Commons Attribution 4.0 International license.

### Correlation analysis of fecal and urinary metabolites, body weight, and bacterial taxa.

The mixOmics (27) package (v.6.10.9) in R was used to conduct correlation analysis. The DIABLO (28) algorithm was used to correlate fecal and urinary metabolites, bacterial taxa at the family level, and phenotypical data (e.g., body weight and body weight changes) in RYGBr and SHAMr group at all time points excluding postantibiotics (D0). The included metabolites and bacterial families are summarized in Table S6. The data from different data sets were matched by animal identifier (IDs) and time points. Lasso-like penalization was employed for feature selection in each data set. The number of components was set at two, and tuning was performed to select the minimum number of features required per data set. A correlation coefficient cutoff of 0.7 was used for data visualization.

### Data availability.

The 16S rRNA sequencing data can be accessed from Sequence Read Archive (SRA) with the BioProject identifier PRJNA675098 or SRA identifier SRP291624 (https://www.ncbi.nlm.nih.gov/sra/PRJNA675098). ^1^H NMR spectral data and R codes used in this study are available in GitHub (https://github.com/jordan129/Rat_Antibiotics_FMT_mSystems_2020).
