# Supplementary figures and images for: A Subset of Roux-en-Y Gastric Bypass Bacterial Consortium Colonizes the Gut of Nonsurgical Rats without Inducing Host-Microbe Metabolic Changes
Source: mSystems. 2020 Dec 8;5(6):e01047-20. doi: 10.1128/mSystems.01047-20 (PMC8579838; doi:10.1128/mSystems.01047-20)

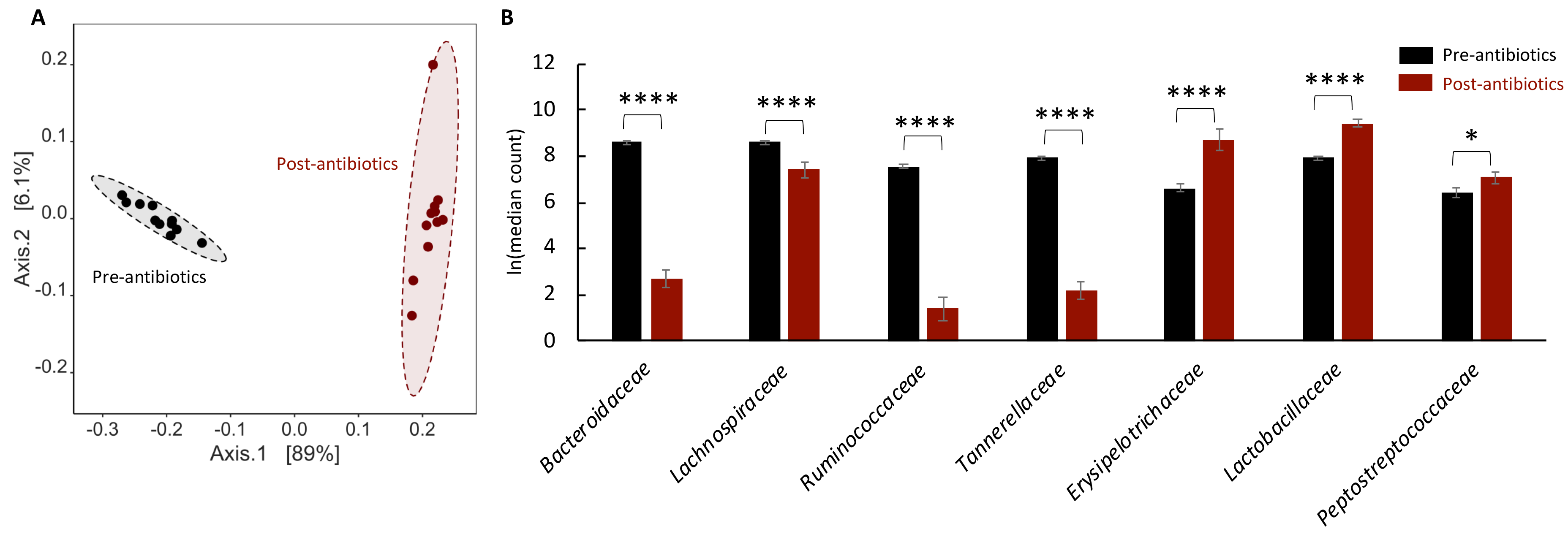

Supplement: FIG S1 [file msystems.01047-20-sf001.tif]

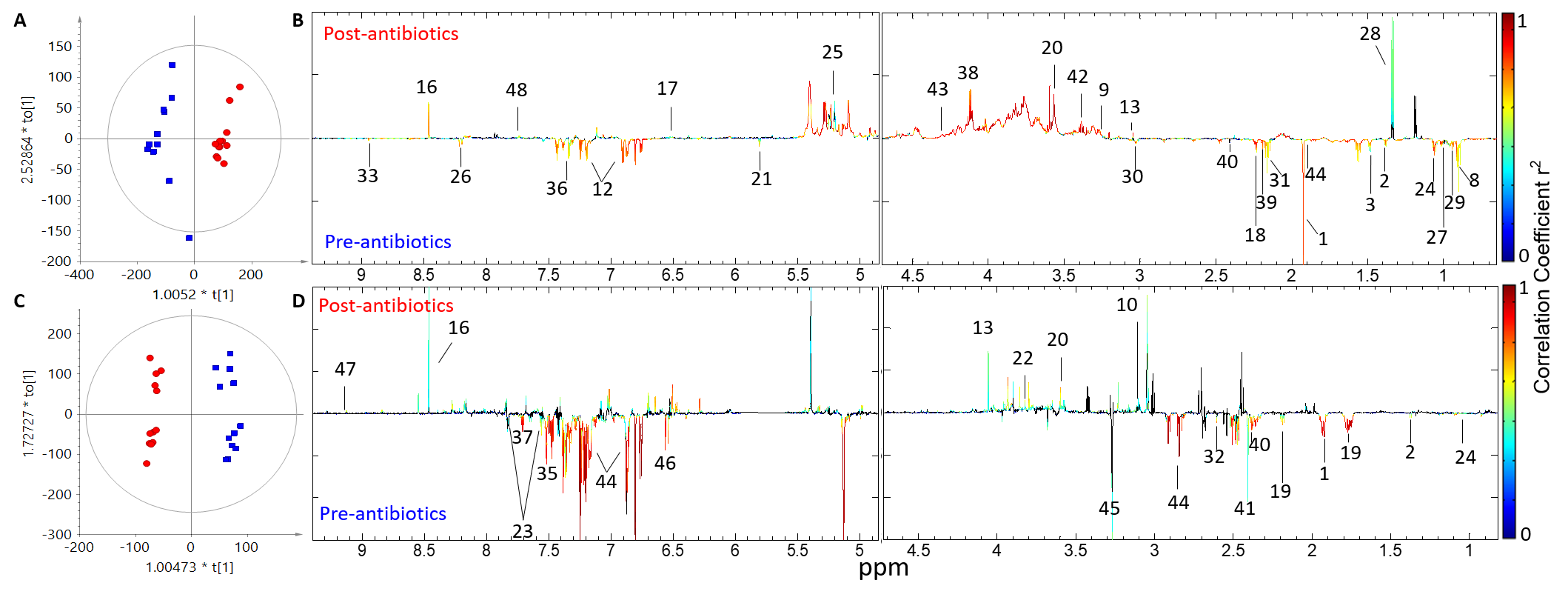

Supplement: FIG S2 [file msystems.01047-20-sf002.tif]

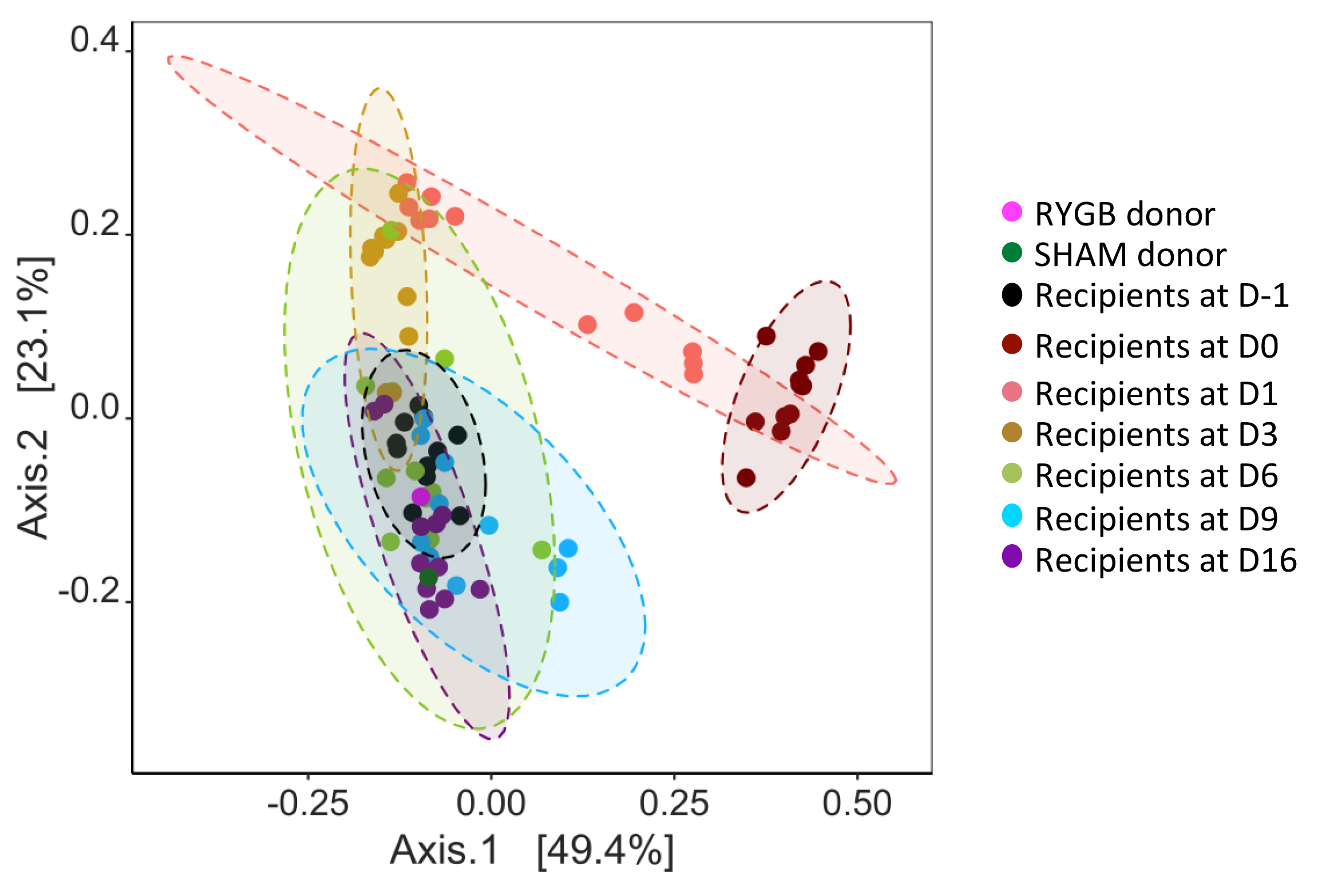

Supplement: FIG S3 [file msystems.01047-20-sf003.tif]

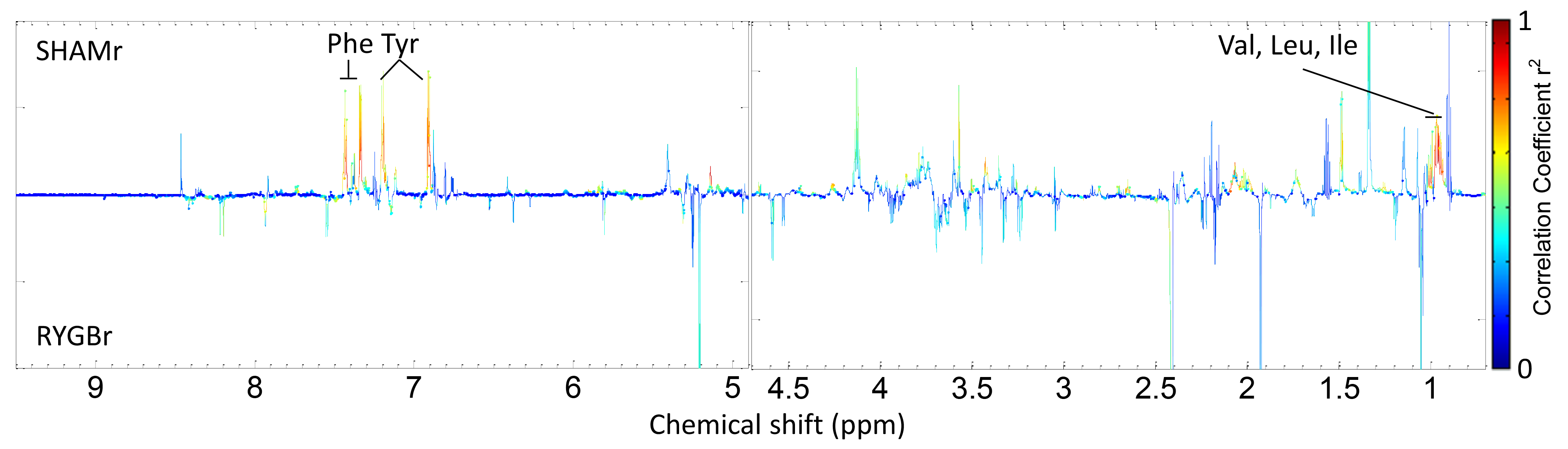

Supplement: FIG S4 [file msystems.01047-20-sf004.tif]
