# Supplementary material for: A Subset of Roux-en-Y Gastric Bypass Bacterial Consortium Colonizes the Gut of Nonsurgical Rats without Inducing Host-Microbe Metabolic Changes
Source: mSystems. 2020 Dec 8;5(6):e01047-20. doi: 10.1128/mSystems.01047-20 (PMC8579838; doi:10.1128/mSystems.01047-20)
Supplement: TABLE S1 [file msystems.01047-20-st001.docx]

| **Key** | **Metabolite** | **δ ^1^H (ppm), Multiplicity & Assignment** | **Changes in feces & r** | **Changes in urine & r** | **Origin** |
| --- | --- | --- | --- | --- | --- |
| 1 | Acetate | 1.92 (s, CH_3_) | ↓ 0.75 | ↓ 0.96 | F, U |
| 2 | Acetoin | 1.37 (s, CH_3_), 2.21 (s, CH_3_), 4.42 (m, CH) | ↓ 0.65 | ↓ 0.76 | F, U |
| 3 | Alanine | 1.48 (d, CH_3_], 3.79 (m, CH_3_) | ↓ 0.53 |  | F, U |
| 4 | Allantoin | 4.43 (q, CH), 2.23 (s), 1.38 (d, CH_3_) | |  | U |
| 5 | alpha-Glucose | 3.25-3.89 (m, 1~5-CH), 4.65 (d, 6-CH2) | ↑ 0.66 |  | F, U |
| 6 | Aspartate | 2.69 (dd), 2.81 (dd), 3.91 (dd) |  |  | F |
| 7 | beta-Glucose | 3.41-3.82 (m, 1~5-CH), 5.24 (d, 6-CH2) | |  | F, U |
| 8 | Butyrate | 2.16 (t, CH_2_), 1.56 (m, CH_2_), 0.89 (t, CH_3_) | ↓ 0.70 |  | F, U |
| 9 | Choline | 3.20 (s, N(CH_3_)_3_) | ↑ 0.86 |  | F, U |
| 10 | cis-Aconitate | 3.11 (d), 5.69 (m) |  | ↑ 0.62 | U |
| 11 | Citrate | 2.55 (d), 2.69 (d) |  |  | U |
| 12 | Tyrosine | 7.19 (d, CH), 6.91 (d, CH) | ↓ 0.72 |  | F |
| 13 | Creatinine | 4.06 (s, CH2), 3.04 (s, CH3) |  | ↑ 0.64 | U |
| 14 | Dimethylamine | 2.72 (s) |  |  | U |
| 15 | Dimethylglycine | 3.71 (s), 2.93 (s) |  |  | U |
| 16 | Formate | 8.46 (s, CH) | ↑ 0.68 | ↑ 0.60 | F, U |
| 17 | Fumarate | 6.52 (s, CH) | ↑ 0.64 |  | F, U |
| 18 | Glutamate | 2.04 (m, CH_2_), 2.36 (m, CH), 3.77 (m) | ↓ 0.53 |  | F |
| 19 | Glutarate | 2.19 (t, CH2), 1.78 (m, CH2) |  | ↓ 0.76 | U |
| 20 | Glycine | 3.57 (s, CH_2_) | ↑ 0.87 | ↑ 0.58 | F, U |
| 21 | Uracil | 7.54 (d, CH), 5.81 (d, CH) | ↓ 0.66 |  | F |
| 22 | Guanidoacetate | 3.80 (s, CH_2_) |  | ↑ 0.79 | U |
| 23 | Hippurate | 7.84 (d, CH_2_), 7.64 (t, CH_2_), 7.56 (t, CH_2_), 3.95 (d, CH_2_) | | ↓ 0.74 | U |
| 24 | Valine | 0.99 (d), 1.04 (d) | ↓ 0.48 | ↓ 0.84 | F, U |
| 25 | Xylose | 5.20 (d), 4.58 (d), 3.92 (dd), 3.6-3.7 (m) | ↑ 0.58 |  | F |
| 26 | Hypoxanthine | 8.19 (s), 8.21 (s) | ↓ 0.79 |  | F |
| 27 | Isoleucine | 1.01 (d, CH_3_), 0.94 (t, CH_3_) | ↓ 0.71 | ↓ 0.56 | F, U |
| 28 | Lactate | 4.12 (m, CH), 1.33 (d, CH­_3_) | ↑ 0.76 |  | F, U |
| 29 | Leucine | 0.96 (d, CH_3_), 0.97 (d, CH3), 1.70 (m, CH_2_) | ↓ 0.61 |  | F, U |
| 30 | Lysine | 3.03 (t, CH_2_), 1.73 (m, CH_2_) | ↓ 0.80 |  | F |
| 31 | Methionine | 2.65 (t, CH_2_), 2.14 (s, CH_3_) | ↓ 0.74 |  | F, U |
| 32 | Methylamine | 2.61 (s, CH_3_) |  | ↓ 0.76 | U |
| 33 | Nicotinate | 8.95 (s, CH), 8.62 (dd, CH), 8.26 (m, CH) | ↓ 0.74 |  | F |
| 34 | 2-Oxoglutarate | 3.01 (t), 2.45 (t) |  |  | U |
| 35 | Phenylacetylglycine | 7.43 (m), 7.36 (m), 3.75 (d) |  | ↓ 0.50 | U |
| 36 | Phenylalanine | 7.43 (t, CH), 7.38 (m, CH), 7.34 (d, CH) | ↓ 0.71 |  | F |
| 37 | 3-Indoxylsulfate | 7.71 (d), 7.51 (d), 7.34 (s), 7.19 (t), 7.152 (t) | | ↓ 0.94 | U |
| 38 | Proline | 2.01 (m, CH_2_), 2.09 (m, CH2), 3.34 (m) | ↑ 0.86 |  | F |
| 39 | Propionate | 2.19 (q, CH_3_), 1.06 (t, CH_2_) | ↓ 0.80 |  | F |
| 40 | Pyruvate | 2.38 (s, CH_3_) | ↑ 0.56 | ↓ 0.83 | F, U |
| 41 | Succinate | 2.41 (s) |  | ↓ 0.57 | F, U |
| 42 | Taurine | 3.27 (t), 3.43 (t) |  |  | U |
| 43 | Threonine | 4.26 (m, CH), 3.59 (d, CH), 1.33 (d, CH_3_) | ↑ 0.84 |  | F |
| 44 | 3-Phenylpropionate | 2.85 (t, CH_2_), 2.48 (t, CH_2_), 7.37 (m, CH), 7.31 (m, CH) | ↓ 0.81 | ↓ 0.96 | F, U |
| 45 | TMAO | 3.27 (s, N(CH_3_)_3_) |  | ↓ 0.86 | U |
| 46 | trans-Aconitate | 6.56 (m), 3.45 (m) |  | ↓ 0.90 | U |
| 47 | Trigonelline | 9.13 (s, CH), 8.84 (t, CH) |  | ↑ 0.76 | U |
| 48 | Tryptophan | 7.74 (d, CH), 7.54 (d) | ↑ 0.68 |  | F |
