# Supplementary material for: A Subset of Roux-en-Y Gastric Bypass Bacterial Consortium Colonizes the Gut of Nonsurgical Rats without Inducing Host-Microbe Metabolic Changes
Source: mSystems. 2020 Dec 8;5(6):e01047-20. doi: 10.1128/mSystems.01047-20 (PMC8579838; doi:10.1128/mSystems.01047-20)
Supplement: TABLE S2 [file msystems.01047-20-st002.docx]

| **Bacterial family/number of taxa** | **Present in RYGB donors & Present in <50% of the recipients at D-1** | **Transferred from RYGB donor only** | | | | | **Present in SHAM donors & Present in <50% of the recipients at D-1** | **Transferred from SHAM donor only** | | | | | **Present in >=50% recipients pre ATB & not present in donors** | **Remained in RYGBr from recipients at D-1** | | | | | **Remained in SHAMr from recipients at D-1** | | | | |
| --- | --- | --- | --- | --- | --- | --- | --- | --- | --- | --- | --- | --- | --- | --- | --- | --- | --- | --- | --- | --- | --- | --- | --- |
|  |  | **D1** | **D3** | **D6** | **D9** | **D16** |  | **D1** | **D3** | **D6** | **D9** | **D16** |  | **D1** | **D3** | **D6** | **D9** | **D16** | **D1** | **D3** | **D6** | **D9** | **D16** |
| *Akkermansiaceae* | 0 | 0 | 0 | 0 | 0 | 0 | 1 | 1 | 1 | 1 | 1 | 1 | 0 | 0 | 0 | 0 | 0 | 0 | 0 | 0 | 0 | 0 | 0 |
| *Atopobiaceae* | 1 | 0 | 0 | 0 | 0 | 0 | 0 | 0 | 0 | 0 | 0 | 0 | 0 | 0 | 0 | 0 | 0 | 0 | 0 | 0 | 0 | 0 | 0 |
| *Bacteroidaceae* | 11 | 2 | 7 | 7 | 6 | 8 | 4 | 0 | 0 | 0 | 0 | 0 | 1 | 1 | 1 | 0 | 0 | 0 | 1 | 2 | 2 | 1 | 1 |
| *Bifidobacteriaceae* | 2 | 1 | 0 | 0 | 0 | 0 | 0 | 0 | 0 | 0 | 0 | 0 | 0 | 0 | 0 | 0 | 0 | 0 | 0 | 0 | 0 | 0 | 0 |
| *Burkholderiaceae* | 10 | 9 | 4 | 3 | 3 | 3 | 7 | 5 | 3 | 3 | 3 | 2 | 0 | 0 | 0 | 0 | 0 | 0 | 0 | 0 | 0 | 0 | 0 |
| *Christensenellaceae* | 0 | 0 | 0 | 0 | 0 | 0 | 2 | 0 | 0 | 0 | 0 | 0 | 3 | 0 | 0 | 1 | 2 | 2 | 0 | 0 | 0 | 1 | 0 |
| *Clostridiaceae_1* | 8 | 0 | 0 | 0 | 0 | 0 | 1 | 1 | 1 | 0 | 1 | 1 | 0 | 0 | 0 | 0 | 0 | 0 | 0 | 0 | 0 | 0 | 1 |
| *Clostridiales_vadinBB60_group* | 10 | 1 | 1 | 2 | 0 | 2 | 2 | 0 | 1 | 0 | 0 | 0 | 0 | 0 | 0 | 0 | 0 | 0 | 0 | 0 | 0 | 0 | 0 |
| *Deferribacteraceae* | 1 | 0 | 0 | 0 | 0 | 0 | 1 | 0 | 0 | 0 | 0 | 0 | 0 | 0 | 0 | 0 | 0 | 0 | 0 | 0 | 0 | 0 | 0 |
| *Desulfovibrionaceae* | 4 | 0 | 2 | 3 | 3 | 3 | 4 | 0 | 2 | 2 | 2 | 0 | 0 | 0 | 0 | 0 | 0 | 0 | 0 | 0 | 0 | 0 | 0 |
| *Eggerthellaceae* | 2 | 0 | 0 | 0 | 0 | 1 | 0 | 0 | 0 | 0 | 0 | 0 | 2 | 0 | 0 | 1 | 1 | 1 | 0 | 0 | 0 | 1 | 0 |
| *Enterobacteriaceae* | 13 | 4 | 1 | 0 | 0 | 0 | 0 | 0 | 0 | 0 | 0 | 0 | 0 | 0 | 0 | 1 | 0 | 0 | 0 | 0 | 0 | 0 | 0 |
| *Enterococcaceae* | 4 | 2 | 1 | 0 | 0 | 0 | 0 | 0 | 0 | 0 | 0 | 0 | 0 | 0 | 0 | 0 | 0 | 0 | 0 | 0 | 0 | 0 | 0 |
| *Erysipelotrichaceae* | 12 | 4 | 9 | 7 | 5 | 1 | 9 | 1 | 4 | 1 | 4 | 3 | 8 | 0 | 1 | 2 | 4 | 1 | 1 | 1 | 3 | 3 | 2 |
| *Eubacteriaceae* | 0 | 0 | 0 | 0 | 0 | 0 | 0 | 0 | 0 | 0 | 0 | 0 | 0 | 0 | 0 | 0 | 0 | 0 | 0 | 0 | 0 | 0 | 0 |
| *Clostridiales Family_XIII* | 3 | 0 | 0 | 2 | 2 | 2 | 8 | 0 | 0 | 2 | 2 | 2 | 1 | 1 | 1 | 0 | 0 | 0 | 1 | 1 | 0 | 0 | 0 |
| *Lachnospiraceae* | 146 | 0 | 3 | 18 | 23 | 42 | 150 | 0 | 2 | 4 | 2 | 4 | 81 | 0 | 2 | 13 | 9 | 14 | 0 | 2 | 6 | 7 | 9 |
| *Lactobacillaceae* | 1 | 0 | 0 | 0 | 0 | 0 | 4 | 2 | 1 | 2 | 2 | 2 | 5 | 5 | 4 | 4 | 4 | 4 | 5 | 5 | 3 | 3 | 3 |
| *Marinifilaceae* | 3 | 0 | 1 | 1 | 1 | 3 | 0 | 0 | 0 | 0 | 0 | 0 | 0 | 0 | 0 | 0 | 0 | 0 | 0 | 0 | 0 | 0 | 0 |
| *Micrococcaceae* | 1 | 0 | 0 | 1 | 0 | 1 | 0 | 0 | 0 | 0 | 0 | 0 | 0 | 0 | 0 | 0 | 0 | 0 | 0 | 0 | 0 | 0 | 0 |
| *Muribaculaceae* | 54 | 0 | 1 | 5 | 7 | 10 | 47 | 0 | 0 | 0 | 2 | 3 | 0 | 0 | 0 | 0 | 0 | 0 | 0 | 0 | 0 | 0 | 0 |
| *Pasteurellaceae* | 1 | 0 | 0 | 0 | 0 | 0 | 0 | 0 | 0 | 0 | 0 | 0 | 0 | 0 | 0 | 0 | 0 | 0 | 0 | 0 | 0 | 0 | 0 |
| *Peptococcaceae* | 3 | 0 | 0 | 0 | 0 | 1 | 6 | 0 | 0 | 0 | 0 | 0 | 1 | 0 | 0 | 1 | 1 | 1 | 0 | 0 | 0 | 0 | 1 |
| *Peptostreptococcaceae* | 1 | 0 | 0 | 0 | 0 | 0 | 1 | 0 | 0 | 0 | 0 | 0 | 4 | 0 | 0 | 0 | 0 | 0 | 0 | 0 | 0 | 0 | 0 |
| *Prevotellaceae* | 5 | 0 | 3 | 4 | 4 | 4 | 5 | 0 | 0 | 1 | 1 | 1 | 0 | 0 | 0 | 0 | 0 | 0 | 0 | 0 | 0 | 0 | 0 |
| *Rikenellaceae* | 13 | 3 | 4 | 7 | 7 | 8 | 6 | 0 | 0 | 1 | 2 | 3 | 0 | 0 | 0 | 0 | 0 | 0 | 0 | 0 | 0 | 0 | 0 |
| *Ruminococcaceae* | 90 | 0 | 1 | 11 | 18 | 35 | 109 | 0 | 0 | 3 | 6 | 15 | 29 | 0 | 3 | 6 | 3 | 2 | 1 | 4 | 4 | 5 | 3 |
| *Saccharimonadaceae* | 1 | 0 | 0 | 0 | 0 | 0 | 1 | 0 | 0 | 0 | 0 | 0 | 0 | 0 | 0 | 0 | 0 | 0 | 0 | 0 | 0 | 0 | 0 |
| *Streptococcaceae* | 3 | 0 | 0 | 0 | 0 | 1 | 0 | 0 | 0 | 0 | 0 | 0 | 0 | 0 | 0 | 0 | 0 | 0 | 0 | 0 | 0 | 0 | 0 |
| *Tannerellaceae* | 6 | 0 | 3 | 4 | 4 | 4 | 4 | 0 | 3 | 3 | 3 | 3 | 1 | 1 | 1 | 0 | 0 | 1 | 1 | 1 | 1 | 1 | 1 |
| *Unclassified_Clostridia* | 0 | 0 | 0 | 0 | 0 | 0 | 0 | 0 | 0 | 0 | 0 | 0 | 1 | 0 | 0 | 0 | 0 | 0 | 0 | 0 | 0 | 0 | 0 |
| *Unclassified_Clostridiales* | 0 | 0 | 0 | 0 | 0 | 0 | 1 | 0 | 0 | 0 | 0 | 0 | 0 | 0 | 0 | 0 | 0 | 0 | 0 | 0 | 0 | 0 | 0 |
| *Unclassified_Coriobacteriales* | 1 | 0 | 0 | 1 | 1 | 1 | 1 | 0 | 0 | 0 | 1 | 1 | 1 | 0 | 0 | 0 | 0 | 0 | 0 | 0 | 0 | 0 | 0 |
| *Unclassified_Gastranaerophilales* | 2 | 0 | 0 | 1 | 1 | 1 | 2 | 0 | 0 | 0 | 0 | 0 | 0 | 0 | 0 | 0 | 0 | 0 | 0 | 0 | 0 | 0 | 0 |
| *Unclassified_Mollicutes_RF39* | 3 | 0 | 0 | 0 | 1 | 1 | 3 | 0 | 0 | 0 | 0 | 0 | 0 | 0 | 0 | 0 | 0 | 0 | 0 | 0 | 0 | 0 | 0 |
| *Unclassified_Rhodospirillales* | 0 | 0 | 0 | 0 | 0 | 0 | 0 | 0 | 0 | 0 | 0 | 0 | 0 | 0 | 0 | 0 | 0 | 0 | 0 | 0 | 0 | 0 | 0 |
| SUM | 415 | 26 | 41 | 77 | 86 | 132 | 379 | 10 | 18 | 23 | 32 | 41 | 138 | 8 | 13 | 29 | 24 | 26 | 10 | 16 | 19 | 22 | 21 |
