# Supplementary material for: A Subset of Roux-en-Y Gastric Bypass Bacterial Consortium Colonizes the Gut of Nonsurgical Rats without Inducing Host-Microbe Metabolic Changes
Source: mSystems. 2020 Dec 8;5(6):e01047-20. doi: 10.1128/mSystems.01047-20 (PMC8579838; doi:10.1128/mSystems.01047-20)
Supplement: TABLE S3 [file msystems.01047-20-st003.docx]

| **ASV ID** | **Taxonomy** | **D1** | **D3** | **D6** | **D9** | **D16** | **Sequences** |
| --- | --- | --- | --- | --- | --- | --- | --- |
| ASV1 | p_Bacteroidetes;c_Bacteroidia;o_Bacteroidales;f_Bacteroidaceae;g_Bacteroides | ↑ |  |  |  |  | GATGAACGCTAGCTACAGGCTTAACACATGCAAGTCGAGGGGCAGCATTTCAGTTTGCTTGCAAACTGGAGATGGCGACCGGCGCACGGGTGAGTAACACGTATCCAACCTGCCGATAACTCGGGGATAGCCTTTCGAAAGAAAGATTAATACCCGATGGTATAATTAGACCGCATGGTCTTGTTATTAAAGAATTTCGGTTATCGATGGGGATGCGTTCCATTAGGCAGTTGGTGAGGTAACGGCTCACCAAACCTTCGATGGATAGGGGTTCTGAGAGGAAGGTCCCCCACATTGGAACTGAGACACGGTCCAA |
| ASV2 | p_Proteobacteria;c_Gammaproteobacteria;o_Betaproteobacteriales;f_Burkholderiaceae;g_Parasutterella | ↑ |  |  |  |  | ATTGAACGCTGGCGGAACGCTTTACACATGCAAGTCGAACGGTAACGCGGGGAGAAGCTTGCTTCTCTCCGGCGACGAGTGGCGAACGGGTGAGTAATACATCGGAACGTGTCCGCTCGTGGGGGACAACCAGCCGAAAGGTTGGCTAATACCGCATGAGTTCTACGGAAGAAAGAGGGGGACCCGCAAGGGCCTCTCGCGAGCGGAGCGGCCGATGACTGATTAGCCGGTTGGTGGGGTAACGGCTCACCAAAGCAACGATCAGTAGCTGGTCTGAGAGGACGACCAGCCACACTGGGACTGAGACACGGCCCAG |
| ASV3 | p_Proteobacteria;c_Gammaproteobacteria;o_Betaproteobacteriales;f_Burkholderiaceae;g_Parasutterella | ↑ |  |  |  |  | ATTGAACGCTGGCGGAACGCTTTACACATGCAAGTCGAACGGTAACGCGGGGAGAAGCTTGCTTCTCTCCGGCGACGAGTGGCGAACGGGTGAGTAATACATCGGAACGTGTCCGCTCGTGGGGGACAACCAGCCGAAAGGTTGGCTAATACCGCATGAGTTCTACGGAAGAAAGAGGGGGACCCGCAAGGGCCTCTCGCGAGCGGAGCGGCCGATGACTGATTAGCCGGTTGGTGAGGTAACGGCTTACCAAAGCAACGATCAGTAGCTGGTCTGAGAGGACGACCAGCCACACTGGGACTGAGACACGGCCCAG |
| ASV4 | p_Bacteroidetes;c_Bacteroidia;o_Bacteroidales;f_Bacteroidaceae;g_Bacteroides |  | ↑ | ↑ |  | ↑ | GATGAACGCTAGCTACAGGCTTAACACATGCAAGTCGAGGGGCAGCATGGTCTTAGCTTGCTAAGGCCGATGGCGACCGGCGCACGGGTGAGTAACACGTATCCAACCTGCCGTCTACTCTTGGACAGCCTTCTGAAAGGAAGATTAATACAAGATGGCATCATGAGTCCGCATGTTCACATGATTAAAGGTATTCCGGTAGACGATGGGGATGCGTTCCATTAGATAGTAGGCGGGGTAACGGCCCACCTAGTCTTCGATGGATAGGGGTTCTGAGAGGAAGGTCCCCCACATTGGAACTGAGACACGGTCCAA |
| ASV5 | p_Bacteroidetes;c_Bacteroidia;o_Bacteroidales;f_Rikenellaceae;g_Alistipes |  | ↑ |  |  |  | GATGAACGCTAGCGGCAGGCTTAACACATGCAAGTCGAGGGGCAGCATATGAGTAGCAATACTTATGATGGCGACCGGCGCACGGGTGCGTAACGCGTATGCAACCTACCTTTTACAGGGGCATAACACTGAGAAATTGGTACTAATTCCCCATAATATCTGAGACGGCATCGTTTTGGGTTGAAAACTCCGGTGGTAAAAGATGGGCATGCGTTGTATTAGCTAGTTGGTGAGGTAACGGCTCACCAAGGCGACGATACATAGGGGGACTGAGAGGTTAACCCCCCACATTGGTACTGAGACACGGACCAA |
| ASV6 | p_Bacteroidetes;c_Bacteroidia;o_Bacteroidales;f_Bacteroidaceae;g_Bacteroides |  | ↑ |  |  |  | GATGAACGCTAGCTACAGGCTTAACACATGCAAGTCGAGGGGCAGCATGGTCTTAGCTTGCTAAGGCCGATGGCGACCGGCGCACGGGTGAGTAACACGTATCCAACCTGCCGTCTACTCTTGGACAGCCTTCTGAAAGGAAGATTAATACAAGATGGCATCATGAGTTCACATGTTCACATGATTAAAGGTATTCCGGTAGACGATGGGGATGCGTTCCATTAGATAGTAGGCGGGGTAACGGCCCACCTAGTCTTCGATGGATAGGGGTTCTGAGAGGAAGGTCCCCCACATTGGAACTGAGACACGGTCCAA |
| ASV7 | p_Firmicutes;c_Erysipelotrichia;o_Erysipelotrichales;f_Erysipelotrichaceae;g_Allobaculum |  | ↑ |  |  |  | GATGAACGCTGGCGGCATGCCTAATACATGCAAGTCGAACGGATATCTTCGGATATCAGTGGCGAACGGGTGAGTAACACGTAGATAACCTGCCCATACCCGGGGGATACGCTTTGGAAACGAAGTCTAACACCCCATAGGAAAGAAGAAGGCATCTTCTTCTTTTGAAACAGGCGTTTGCCTGGGGGACGGATGGATCTGCGGTGCATTAGTTAGTTGGCGAGGTAACAGCTCACCAAGACGATGATGCATAGCCGGCCTGAGAGGGCGATCGGCCACACTGGGACTGAGACACGGCCCAG |
| ASV8 | p_Proteobacteria;c_Gammaproteobacteria;o_Betaproteobacteriales;f_Burkholderiaceae;g_Parasutterella |  | ↓ |  |  |  | ATTGAACGCTGGCGGAACGCTTTACACATGCAAGTCGAACGGTAACGCGGAGAGAAGCTTGCTTCTCTCCGGCGACGAGTGGCGAACGGGTGAGTAATACATCGGAACGTGTCCGCTCGTGGGGGACAACCAGCCGAAAGGTTGGCTAATACCGCATGAGTTCTACGGAAGAAAGAGGGGGACCCGCAAGGGCCTCTCGCGAGCGGAGCGGCCGATGACTGATTAGCCTGTTGGTGAGGTAACGGCTCACCAAAGCAACGATCAGTAGCTGGTCTGAGAGGACGACCAGCCACACTGGGACTGAGACACGGCCCAG |
| ASV9 | p_Bacteroidetes;c_Bacteroidia;o_Bacteroidales;f_Rikenellaceae;g_Alistipes |  |  | ↑ |  |  | GATGAACGCTAGCGGCAGGCTTAACACATGCAAGTCGAGGGGCAGCACGAGGTAGCAATACTTTGGTGGCGACCGGCGCACGGGTGCGTAACGCGTATGCAACCTACCTTTAACAGGGGCATAACACTGAGAAATTGGTACTAATTCCCCATAACATTCGAGAAGGCATCTTCTTGGGTTAAAAACTCCGGTGGTTAAAGATGGGCATGCGTTGTATTAGCTAGTTGGTGAGGTAACGGCTCACCAAGGCAACGATACATAGGGGGACTGAGAGGTTAACCCCCCACATTGGTACTGAGACACGGACCAA |
| ASV10 | p_Proteobacteria;c_Deltaproteobacteria;o_Desulfovibrionales;f_Desulfovibrionaceae;g_Bilophila |  |  | ↓ |  |  | ATTGAACGCTGGCGGCGTGCTTAACACATGCAAGTCGAACGTGAAAGTCCTTCGGGACGAGTAAAGTGGCGCACGGGTGAGTAACGCGTGGATAATCTACCCTTAAGATGGGGATAACGGCTGGAAACGGTCGCTAATACCGAATACGCTCCCGATTTTATCGTTGGGGGGAAAGATGGCCTCTGCTTGCAAGCTATCGCTTAAGGATGAGTCCGCGTCCCATTAGCTAGTTGGCGGGGTAACGGCCCACCAAGGCGACGATGGGTAGCCGGTCTGAGAGGATGACCGGCCACACTGGAACTGGAACACGGTCCAG |
| ASV11 | p_Firmicutes;c_Clostridia;o_Clostridiales;f_Lachnospiraceae;g_Unclassified |  |  | ↓ |  |  | GATGAACGCTGGCGGCGTGCTTAACACATGCAAGTCGAACGAAGCGCCTGCTTTGAATTCCTACGGGAAGGAGAGGCTTGCGACTGAGTGGCGGACGGGTGAGTAACGCGTGGGCAACCTGCCCCGCACTGGGGGACAACAGCTGGAAACGGCTGCTAATACCGCATAAGCGCACAGCTTCGCATGAAGCGGTGTGAAAAACTCCGGTGGTGCGGGATGGGCCCGCGTCTGATTAGCTGGTTGGCGGGGTAACGGCCCACCAAGGCAACGATCAGTAGCCGGCCTGAGAGGGTGGACGGCCACATTGGGACTGAGACACGGCCCAG |
| ASV12 | p_Firmicutes;c_Clostridia;o_Clostridiales;f_Ruminococcaceae;g_NK4A214_group |  |  | ↓ | ↓ | ↓ | GATGAACGCTGGCGGCGTGCCTAACACATGCAAGTCGAACGAAGCACTTCTGATCGGAGATTCGTCAAAGATTGGATTTGACTTAGTGGCGGACGGGTGAGTAACGCGTGAGCAACCTGCCTTTCAGAGGGGGACAACAGTTGGAAACGACTGCTAATACCGCATGATGTATTTAGGAGGCATCTTCTGAATACCAAAGGAGCAATCCGCTGAAAGATGGGCTCACGTCTGATTAGCTAGTTGGTGAGGTAACGGCTCACCAAGGCTGCGATCAGTAGCCGGACTGAGAGGTTGAACGGCCACATTGGGACTGAGATACGGCCCAG |
| ASV13 | p_Firmicutes;c_Clostridia;o_Clostridiales;f_Ruminococcaceae;g_Ruminococcus_1 |  |  |  | ↑ |  | GACGAACGCTGGCGGCACGCCTAACACATGCAAGTCGAACGGACGAGGAGGAGCTTGCTTCTCCGAGTTAGTGGCGGACGGGTGAGTAACACGTGAGCAACCTGCCCTTGAGAGGGGGATAGCTTCTGGAAACGGATGGTAATACCCCATAACATATATTTTAGGCATCTAAGATATATCAAAGAAATTCGCTCAAGGATGGGCTCGCGTCTGATTAGATAGTTGGTGAGGTAACGGCCCACCAAGTCGACGATCAGTAGCCGGACTGAGAGGTTGAACGGCCACATTGGGACTGAGACACGGCCCAG |
| ASV14 | p_Firmicutes;c_Clostridia;o_Clostridiales;f_Ruminococcaceae;g_Unclassified |  |  |  | ↑ |  | GATGAACGCTGGCGGCACGCCTAACACATGCAAGTCGAACGAGGTTATTATGATTGAAGCCTTCGGGCGGATTGAGTTTTAACCTAGTGGCGGACGGGTGAGTAACACGTGAGCAATCTGCCTTTCAGAGCGGGATACCGTTTGGAAACGAACGTTAATACCGCATAACGTAACGTTACCGCATGGTTTTGTTACCAAAGATTTTATCGCTGAAAGATGAGCTCGCGTCTGATTAGATAGTTGGTGAGGTAACGGCCCACCAAGTCGACGATCAGTAGCCGGACTGAGAGGTTGAACGGCCACATTGGGACTGAGACACGGCCCAG |
| ASV15 | p_Firmicutes;c_Bacilli;o_Lactobacillales;f_Lactobacillaceae;g_Lactobacillus |  |  |  | ↓ |  | GATGAACGCCGGCGGTGTGCCTAATACATGCAAGTCGTACGCACTGGCCCAACTGATTGATGGTGCTTGCACCTGATTGACGATGGATCACCAGTGAGTGGCGGACGGGTGAGTAACACGTAGGTAACCTGCCCCGGAGCGGGGGATAACATTTGGAAACAGATGCTAATACCGCATAACAACAAAAGCCACATGGCTTTTGTTTGAAAGATGGCTTTGGCTATCACTCTGGGATGGACCTGCGGTGCATTAGCTAGTTGGTAAGGTAACGGCTTACCAAGGCGATGATGCATAGCCGAGTTGAGAGACTGATCGGCCACAATGGAACTGAGACACGGTCCAT |
| ASV16 | p_Firmicutes;c_Bacilli;o_Lactobacillales;f_Lactobacillaceae;g_Lactobacillus |  |  |  | ↓ |  | GACGAACGCTGGCGGCGTGCCTAATACATGCAAGTCGAGCGAGCTGAACCAGCAGATTCACTTCGGTGATGACGCTGGGAACGCGAGCGGCGGATGGGTGAGTAACACGTGGGTAACCTGCCCTAAAGTCTGGGATACCACTTGGAAACAGGTGCTAATACCGGATAACAACAATAGCTGCATGGCTATTGCTTAAAAGGCGGCGAAAGCTGTCGCTAAAGGATGGACCCGCGGTGCATTAGCTAGTTGGTAAGGTAATGGCTTACCAAGGCGACGATGCATAGCCGAGTTGAGAGACTGATCGGCCACATTGGGACTGAGACACGGCCCAA |
| ASV17 | p_Bacteroidetes;c_Bacteroidia;o_Bacteroidales;f_Tannerellaceae;g_Parabacteroides |  |  |  |  | ↑ | GATGAACGCTAGCGACAGGCTTAACACATGCAAGTCGAGGGGCAGCGGAGTGTAGCAATACATTGCCGGCGACCGGCGCACGGGTGAGTAACGCGTATGCAACTTACCTATCAGAGGGGAATAGCCCGGCGAAAGTCGGATTAATGCCCCATAAAACAGGGGTCCCGCATGGGACTATTTGTTAAAGATTCATCGCTGATAGATAGGCATGCGTTCCATTAGGCAGTTGGCGGGGTAACGGCCCACCAAACCGACGATGGATAGGGGTTCTGAGAGGAAGGTCCCCCACATTGGAACTGAGACACGGTCCAA |
| ASV18 | p_Firmicutes;c_Clostridia;o_Clostridiales;f_Ruminococcaceae;g_Ruminococcaceae_UCG-014 |  |  |  |  | ↑ | GATAAACGCTGGCGGCGCACATAAGACATGCAAGTCGAACGGACTTAACTCATTCTTTTAGTTTGAGAGCGGTTAGTGGCGGACTGGTGAGTAACACGTAAGCAACCTGCCTATCAGAGGGGAATAACAACGAGAAATCGTTGCTAATACCGCATATGCTCACAGTACCACATGGTACAGTGAGGAAAGGAGCAATCCGCTGATAGATGGGCTTGCGTCTGATTAGCTAGTTGGTGGGGTAACGGCCTACCAAGGCAACGATCAGTAGCCGGACTGAGAGGTTGAACGGCCACATTGGGACTGAGATACGGCCCAG |
| ASV19 | p_Firmicutes;c_Clostridia;o_Clostridiales;f_Ruminococcaceae;g_Ruminiclostridium_5 |  |  |  |  | ↑ | GACGAACGCTGGCGGCGTGCCTAACACATGCAAGTCGAACGGAGTGTTACGCTGAACGGAAGGTGCTTGCACTGGAAGTTCTTGTAACACTTAGTGGCGGACGGGTGAGTAACGCGTGAGCAACCTGCCTTTCAGAGGGGGATAACGTTTGGAAACGAACGCTAATACCGCATGAGACTACGGTACCGCATGGTAGAGTGGTCAAAGGAGCAATCCGCTGAAAGATGGGCTCGCGTCCGATTAGATAGTTGGCGGGGTAGAGGCCCACCAAGTCGACGATCGGTAGCCGGACTGAGAGGTTGAACGGCCACATTGGGACTGAGACACGGCCCAG |
| ASV20 | p_Firmicutes;c_Clostridia;o_Clostridiales;f_Lachnospiraceae;g_NK4A136_group |  |  |  |  | ↑ | GATGAACGCTGGCGGCGTGCTTAACACATGCAAGTCGAACGGGGTTATCATTTTTGAAGCGATTAGCTTGCTAAGAGTGGAAATGTTGATAACTTAGTGGCGGACGGGTGAGTAACGCGTGGGTAACCTGCCATATACAGGGGGATAACACTTAGAAATAGGTGCTAATACCGCATAAGCGCACAGAGCTGCATGGCTCAGTGTGAAAAACTCCGGTGGTATATGATGGACCCGCGTCTGATTAGCTTGTTGGCGGGGTAACGGCCCACCAAGGCGACGATCAGTAGCCGGCCTGAGAGGGTGGACGGCCACATTGGGACTGAGACACGGCCCAA |
