# Supplementary material for: A Subset of Roux-en-Y Gastric Bypass Bacterial Consortium Colonizes the Gut of Nonsurgical Rats without Inducing Host-Microbe Metabolic Changes
Source: mSystems. 2020 Dec 8;5(6):e01047-20. doi: 10.1128/mSystems.01047-20 (PMC8579838; doi:10.1128/mSystems.01047-20)
Supplement: TABLE S6 [file msystems.01047-20-st006.docx]

| **Bacteria Family** | **Fecal metabolites** | **Urine metabolites** |
| --- | --- | --- |
| Bacteroidaceae | acetate | methylnicotinamide |
| Lactobacillaceae | acetoin | 2-oxoglutarate |
| Lachnospiraceae | alanine | hydroxybutyrate |
| Erysipelotrichaceae | aspartate | indoxylsulfate |
| Ruminococcaceae | butyrate | hydroxyphenylacetate |
| Tannerellaceae | choline | acetate |
| Rikenellaceae | formate | acetoin |
| Peptostreptococcaceae | fumarate | alanine |
| Burkholderiaceae | glutamate | allantoin |
| Prevotellaceae | glycine | alpha_glucose |
| Muribaculaceae | hypoxanthine | beta_glucose |
| Akkermansiaceae | isoleucine | butyrate |
| Christensenellaceae | lactate | choline |
| Desulfovibrionaceae | leucine | cis_aconitate |
| Clostridiaceae_1 | lysine | citrate |
| Enterobacteriaceae | methionine | creatine |
| Peptococcaceae | nicotinate | creatinine |
| Clostridialesamily_XIII | phenylalanine | dimethylamine |
| Deferribacteraceae | proline | dimethylglycine |
| Eggerthellaceae | propionate | formate |
| Clostridiales_vadinBB60_group | pyruvate | fumarate |
| Unclassified_Gastranaerophilales | succinate | glutarate |
| Unclassified_Rhodospirillales | threonine | glycine |
| Marinifilaceae | tryptophan | glycerophosphocholine |
| Bifidobacteriaceae | tyrosine | guanidoacetate |
| Enterococcaceae | uracil | hippurate |
| Unclassified_Mollicutes_RF39 | valine | isoleucine |
|  | xylose | lactate |
|  | alpha_glucose | leucine |
|  | beta_glucose | methionine |
|  | phenylpropionate | methylamine |
|  |  | nicotinurate |
|  |  | phenylacetylglycine |
|  |  | pyruvate |
|  |  | succinate |
|  |  | taurine |
|  |  | Trimethylamine *N*-oxide |
|  |  | trans_aconitate |
|  |  | trigonelline |
|  |  | tyrosine |
|  |  | valine |
