# Supplementary material for: A Subset of Roux-en-Y Gastric Bypass Bacterial Consortium Colonizes the Gut of Nonsurgical Rats without Inducing Host-Microbe Metabolic Changes
Source: mSystems. 2020 Dec 8;5(6):e01047-20. doi: 10.1128/mSystems.01047-20 (PMC8579838; doi:10.1128/mSystems.01047-20)
Supplement: TABLE S4 [file msystems.01047-20-st004.docx]

| Forward (These primers are mixed at a 4:1:1:1 ratio (28F-YM is the 4) | |
| --- | --- |
| 28F-YM | **TCGTCGGCAGCGTCAGATGTGTATAAGAGACAG**GAGTTTGATYMTGGCTCAG |
| 28F-Borrellia | **TCGTCGGCAGCGTCAGATGTGTATAAGAGACAG**GAGTTTGATCCTGGCTTAG |
| 28FChloroflex | **TCGTCGGCAGCGTCAGATGTGTATAAGAGACAG**GAATTTGATCTTGGTTCAG |
| 28F-Bifdo | **TCGTCGGCAGCGTCAGATGTGTATAAGAGACAG**GGGTTCGATTCTGGCTCAG |
| Reverse | |
| 388R: | **GTCTCGTGGGCTCGGAGATGTGTATAAGAGACAG**TGCTGCCTCCCGTAGGAGT |
