# Supplementary material for: A Subset of Roux-en-Y Gastric Bypass Bacterial Consortium Colonizes the Gut of Nonsurgical Rats without Inducing Host-Microbe Metabolic Changes
Source: mSystems. 2020 Dec 8;5(6):e01047-20. doi: 10.1128/mSystems.01047-20 (PMC8579838; doi:10.1128/mSystems.01047-20)
Supplement: TABLE S5 [file msystems.01047-20-st005.docx]

| **SampleID** | **Group** | **Filtered reads** | **Rarefied reads** |
| --- | --- | --- | --- |
| RYGB-donor | RYGB_donor | 21914 | 16265 |
| 12-pre-ATB | RYGBr_D-1 | 23496 | 16265 |
| 17-pre-ATB | RYGBr_D-1 | 16293 | 16265 |
| 1-pre-ATB | RYGBr_D-1 | 23507 | 16265 |
| 22-pre-ATB | RYGBr_D-1 | 23741 | 16265 |
| 4-pre-ATB | RYGBr_D-1 | 24035 | 16265 |
| 8-pre-ATB | RYGBr_D-1 | 18356 | 16265 |
| 12-post-ATB | RYGBr_D0 | 25329 | 16265 |
| 17-post-ATB | RYGBr_D0 | 19359 | 16265 |
| 1-post-ATB | RYGBr_D0 | 22303 | 16265 |
| 22-post-ATB | RYGBr_D0 | 19579 | 16265 |
| 4-post-ATB | RYGBr_D0 | 22988 | 16265 |
| 8-post-ATB | RYGBr_D0 | 21306 | 16265 |
| 12-post-D1 | RYGBr_D1 | 27577 | 16265 |
| 17-post-D1 | RYGBr_D1 | 22562 | 16265 |
| 1-post-D1 | RYGBr_D1 | 27775 | 16265 |
| 22-post-D1 | RYGBr_D1 | 27972 | 16265 |
| 4-post-D1 | RYGBr_D1 | 26071 | 16265 |
| 8-post-D1 | RYGBr_D1 | 21068 | 16265 |
| 12-post-D3 | RYGBr_D3 | 19793 | 16265 |
| 17-post-D3 | RYGBr_D3 | 22088 | 16265 |
| 1-post-D3 | RYGBr_D3 | 27276 | 16265 |
| 22-post-D3 | RYGBr_D3 | 24119 | 16265 |
| 4-post-D3 | RYGBr_D3 | 25218 | 16265 |
| 8-post-D3 | RYGBr_D3 | 24763 | 16265 |
| 12-post-D6 | RYGBr_D6 | 23258 | 16265 |
| 17-post-D6 | RYGBr_D6 | 23418 | 16265 |
| 1-post-D6 | RYGBr_D6 | 24167 | 16265 |
| 22-post-D6 | RYGBr_D6 | 29195 | 16265 |
| 4-post-D6 | RYGBr_D6 | 22546 | 16265 |
| 8-post-D6 | RYGBr_D6 | 22216 | 16265 |
| 12-post-D9 | RYGBr_D9 | 19365 | 16265 |
| 17-post-D9 | RYGBr_D9 | 18552 | 16265 |
| 1-post-D9 | RYGBr_D9 | 26570 | 16265 |
| 22-post-D9 | RYGBr_D9 | 23028 | 16265 |
| 4-post-D9 | RYGBr_D9 | 24440 | 16265 |
| 8-post-D9 | RYGBr_D9 | 19059 | 16265 |
| 12-post-D16 | RYGBr_D16 | 20215 | 16265 |
| 17-post-D16 | RYGBr_D16 | 17227 | 16265 |
| 1-post-D16 | RYGBr_D16 | 25634 | 16265 |
| 22-post-D16 | RYGBr_D16 | 20695 | 16265 |
| 4-post-D16 | RYGBr_D16 | 28738 | 16265 |
| 8-post-D16 | RYGBr_D16 | 37488 | 16265 |
| Sham-donor | Sham_donor | 21827 | 16265 |
| 10-pre-ATB | SHAMr_D-1 | 19679 | 16265 |
| 11-pre-ATB | SHAMr_D-1 | 18522 | 16265 |
| 13-pre-ATB | SHAMr_D-1 | 23258 | 16265 |
| 19-pre-ATB | SHAMr_D-1 | 22194 | 16265 |
| 5-pre-ATB | SHAMr_D-1 | 18995 | 16265 |
| 10-post-ATB | SHAMr_D0 | 18850 | 16265 |
| 11-post-ATB | SHAMr_D0 | 32650 | 16265 |
| 13-post-ATB | SHAMr_D0 | 19836 | 16265 |
| 19-post-ATB | SHAMr_D0 | 22420 | 16265 |
| 5-post-ATB | SHAMr_D0 | 16265 | 16265 |
| 10-post-D1 | SHAMr_D1 | 27999 | 16265 |
| 11-post-D1 | SHAMr_D1 | 25183 | 16265 |
| 13-post-D1 | SHAMr_D1 | 31606 | 16265 |
| 19-post-D1 | SHAMr_D1 | 28593 | 16265 |
| 5-post-D1 | SHAMr_D1 | 25034 | 16265 |
| 10-post-D3 | SHAMr_D3 | 21645 | 16265 |
| 11-post-D3 | SHAMr_D3 | 21346 | 16265 |
| 13-post-D3 | SHAMr_D3 | 24512 | 16265 |
| 19-post-D3 | SHAMr_D3 | 24887 | 16265 |
| 5-post-D3 | SHAMr_D3 | 22975 | 16265 |
| 10-post-D6 | SHAMr_D6 | 26892 | 16265 |
| 11-post-D6 | SHAMr_D6 | 23747 | 16265 |
| 13-post-D6 | SHAMr_D6 | 27025 | 16265 |
| 19-post-D6 | SHAMr_D6 | 32337 | 16265 |
| 5-post-D6 | SHAMr_D6 | 18613 | 16265 |
| 10-post-D9 | SHAMr_D9 | 19999 | 16265 |
| 11-post-D9 | SHAMr_D9 | 19723 | 16265 |
| 13-post-D9 | SHAMr_D9 | 17921 | 16265 |
| 19-post-D9 | SHAMr_D9 | 19292 | 16265 |
| 5-post-D9 | SHAMr_D9 | 29692 | 16265 |
| 10-post-D16 | SHAMr_D16 | 22346 | 16265 |
| 11-post-D16 | SHAMr_D16 | 26368 | 16265 |
| 13-post-D16 | SHAMr_D16 | 20521 | 16265 |
| 19-post-D16 | SHAMr_D16 | 21126 | 16265 |
| 5-post-D16 | SHAMr_D16 | 35923 | 16265 |
